# Supplementary material for: Transcription Factor Levels after Forward Programming of Human Pluripotent Stem Cells with GATA1, FLI1, and TAL1 Determine Megakaryocyte versus Erythroid Cell Fate Decision
Source: Stem Cell Reports. 2018 Nov 29;11(6):1462–78. doi: 10.1016/j.stemcr.2018.11.001 (PMC6294717; doi:10.1016/j.stemcr.2018.11.001)
Supplement: Document S1. Supplemental Experimental Procedures, Figures S1–S6, and Tables S1 and S2 [file mmc1.pdf]

Stem Cell Reports, Volume *11*

Supplemental Information

Transcription Factor Levels after Forward Programming of Human Pluripotent Stem Cells with GATA1, FLI1, and TAL1 Determine Megakaryocyte versus Erythroid Cell Fate Decision

Amanda Dalby, Jose Ballester-Beltrán, Chiara Lincetto, Annett Mueller, Nicola Foad, Amanda Evans, James Baye, Ernest Turro, Thomas Moreau, Marloes R. Tijssen, and Cedric Ghevaert

Supplementary Figures

Supplementary Figure 1: Mixed effect logistic regression analysis of the expression of CD41a and CD235a of forward programmed cells

A. Day 9 post-transduction

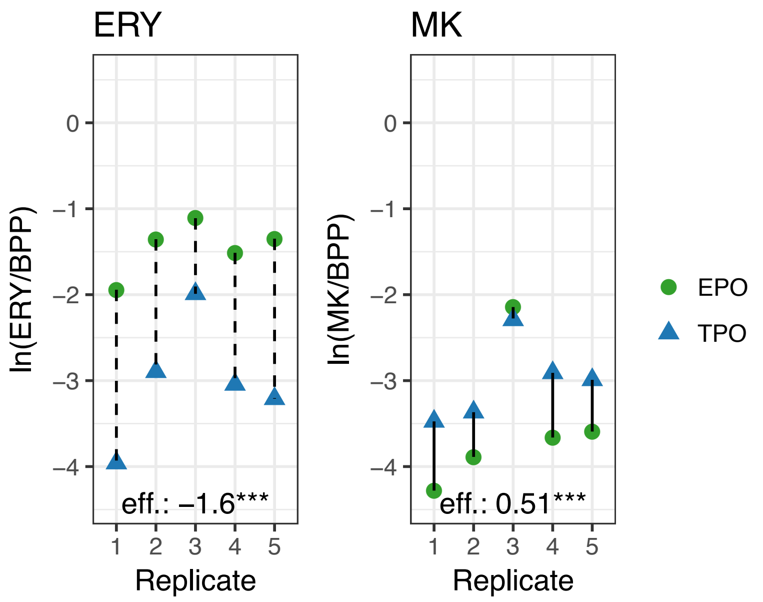

B. Day 20 post-transduction

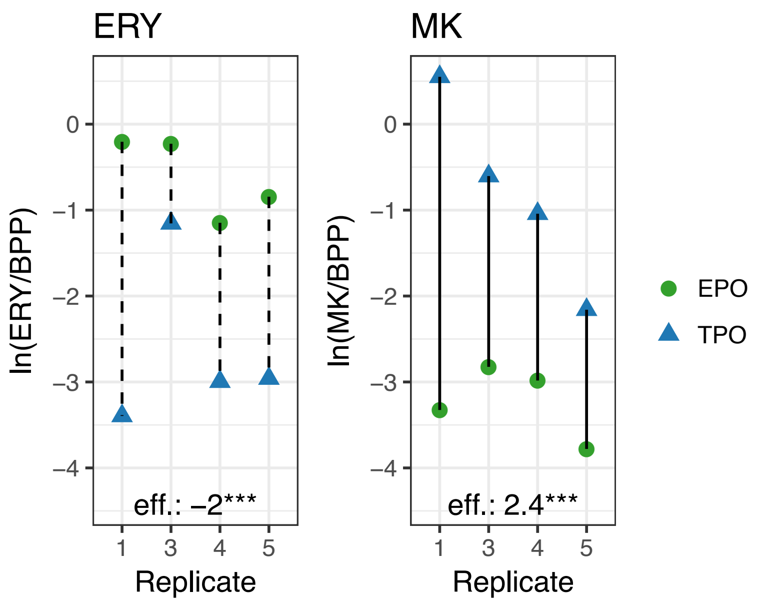

C. Cytokine Switch Experiment

First treatment held constant | **Second treatment held constant**

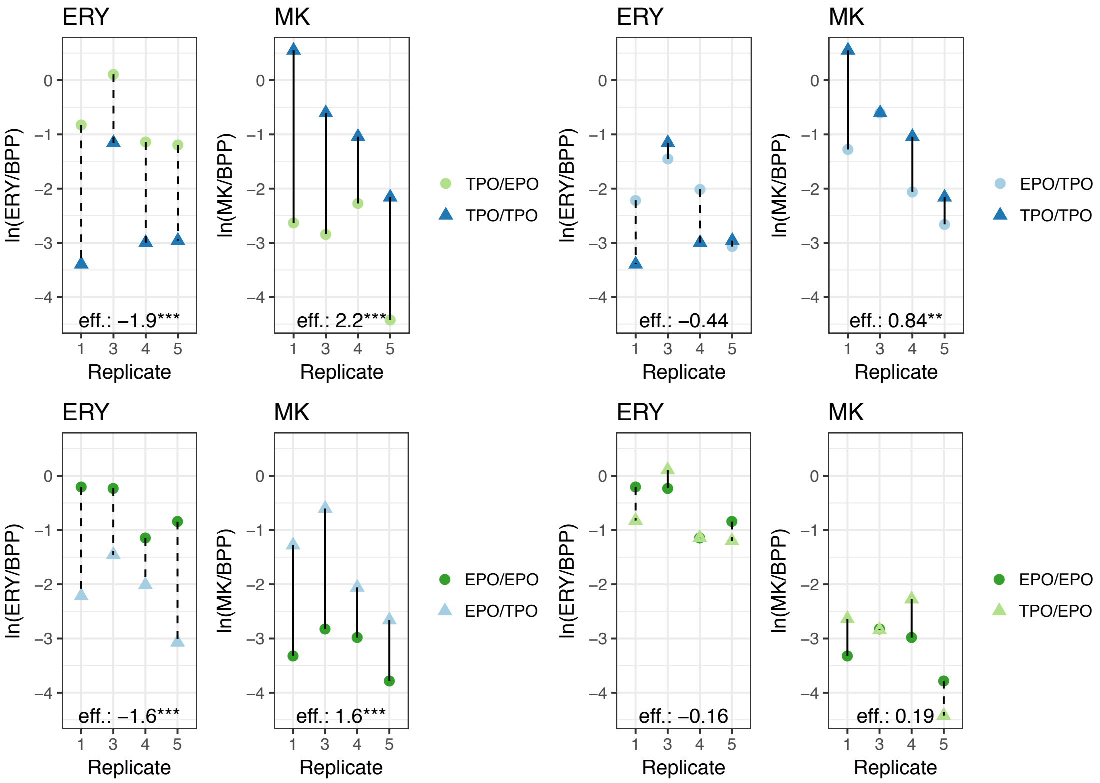

Supplementary Figure 1. Mixed effect logistic regression analysis of the expression of CD41a and CD235a of forward programmed cells

The flow cytometry data were analysed using mixed effects logistic regression. The binary outcome indicated whether a cell was CD41+CD235- (MK) or CD41+CD235+ (BPP) in one analysis and CD41-CD235+ (ERY) or CD41+CD235+ (BPP) in another analysis. This was performed for each replicate experiment of the cells harvested at day 9 (**A**), day 20 (**B**) where the same cytokine was used from day 2 till day 20 and for the cytokine switch experiment (**C**) where cytokine were switched at day 9 and data analysed at day 20. For each replicate the log(e) of the numbers of ERY or MK over the number of BPP is represented and color-coded according to the cytokine the cells were grown in. The cytokine “effect” is represented by the vertical line between the two cytokine conditions in each replicate. The models included a fixed effect for each replicate and random slopes with common variance representing the replicate-specific changes in the log odds due to cytokine exposure (e.g. TPO vs EPO). The random slopes accounted for evident variation in the treatment effects across replicates.

Supplementary Figure 2. CFU-assay colony characterization for myeloid lineages.

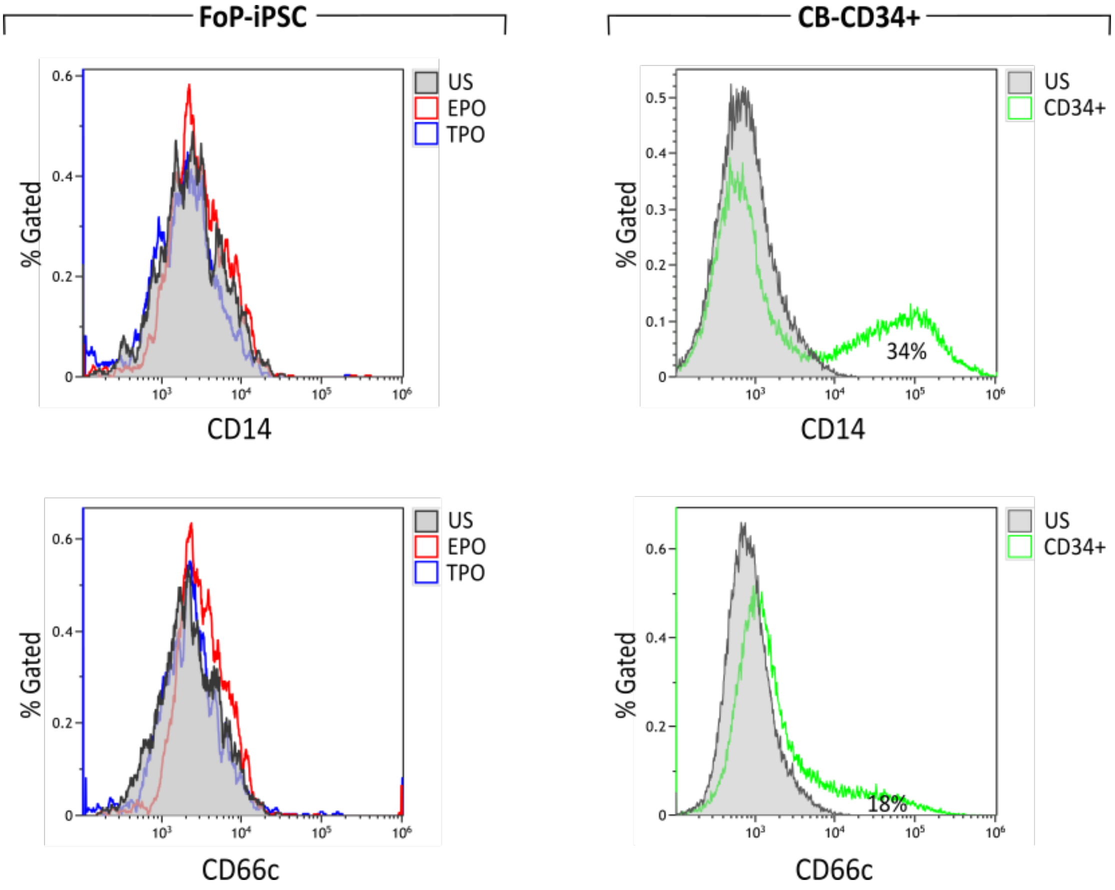

Supplementary Figure 2. CFU-assay colony characterization for myeloid lineages. The whole colony content of CFU assays was analysed by flow cytometry for CD14 (monocyte) and CD66c (granulocyte) expression, from day 9 forward programmed cells grown in EPO (red line) and TPO (blue line), or from CD34+ primary cord blood progenitors (green line); unstained cells (grey line).

Supplementary figure 3. Forward programming of additional hPSC lines to produce erythroblasts.

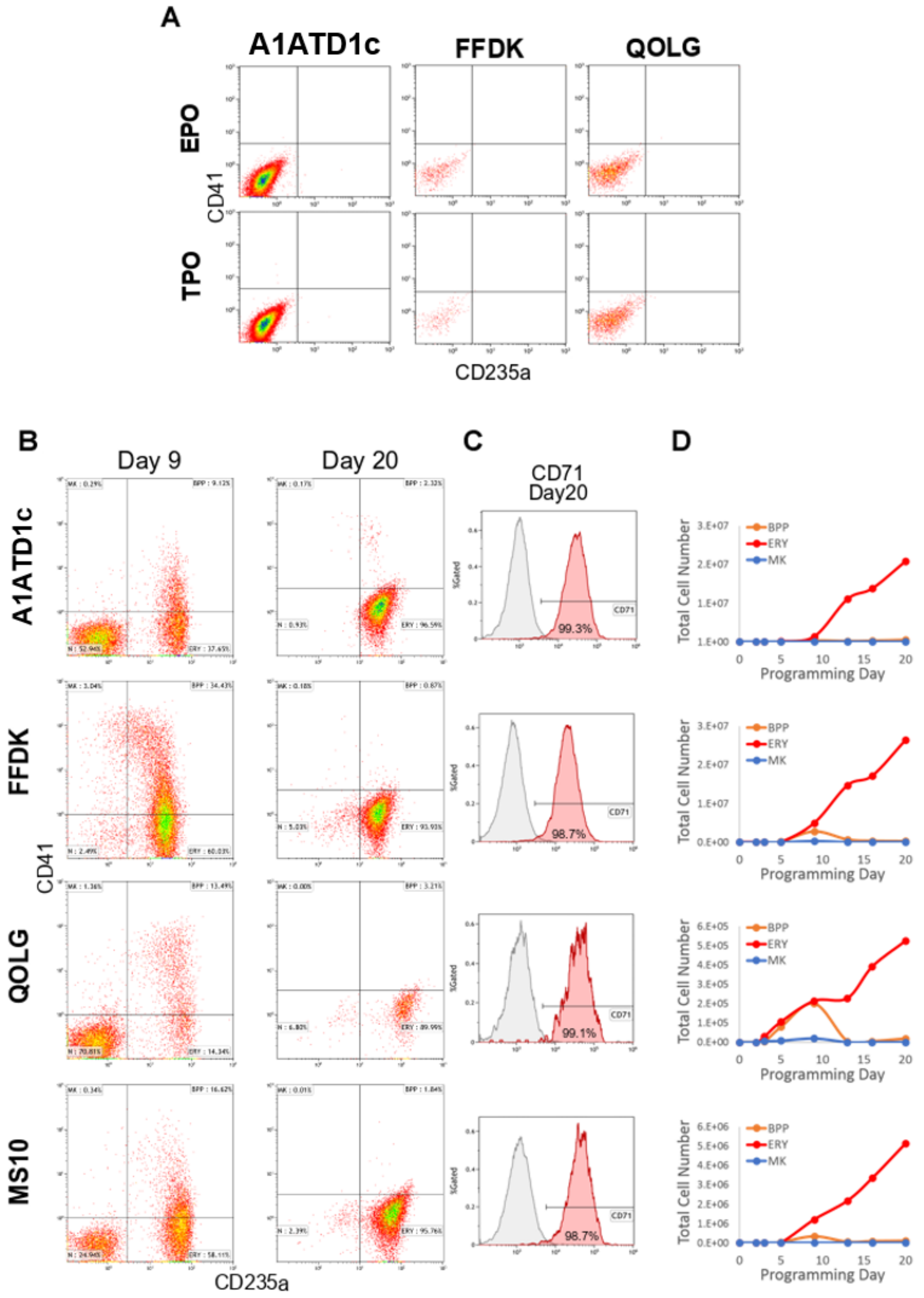

Supplementary figure 3. Forward programming of additional hPSC lines to produce erythroblasts. In addition to the iPSC line presented in the main figure (A1ATD1c), an additional 2 iPSC lines (FFDK and QOLG) and an ESC line were forward programmed with the 3TFs GATA1, TAL1 and FLI1 in EPO culture conditions. **(A)** Representative dot plots of untransduced cells for the expression of CD41a and CD235a after 9 days in EPO culture showing no evidence of cell differentiation towards the erythroid or MK lineage. **(B)** Representative flow cytometry dot plots for CD41a and CD235a expression on day 9 (left) and day 20 of culture (right) showing the presence of a population of cells expressing both markers at day 9 as well as single positive CD235a ERYs whilst the day 20 cells represent a pure ERY population. **(C)** Flow cytometry histogram showing CD71

expression of the forward programmed cells at day 20. **(D)** Graphs demonstrating the cell numbers accumulating over time according to cell type: BPP (CD235a+ CD41a+), ERY (CD235a+ CD41a-) and MKs (CD235a- CD41a+) starting from $3x10^4$ stem cells.

Supplementary Figure 4. Clonogenic assay with forward programmed cells seeded at day 5 post-transduction.

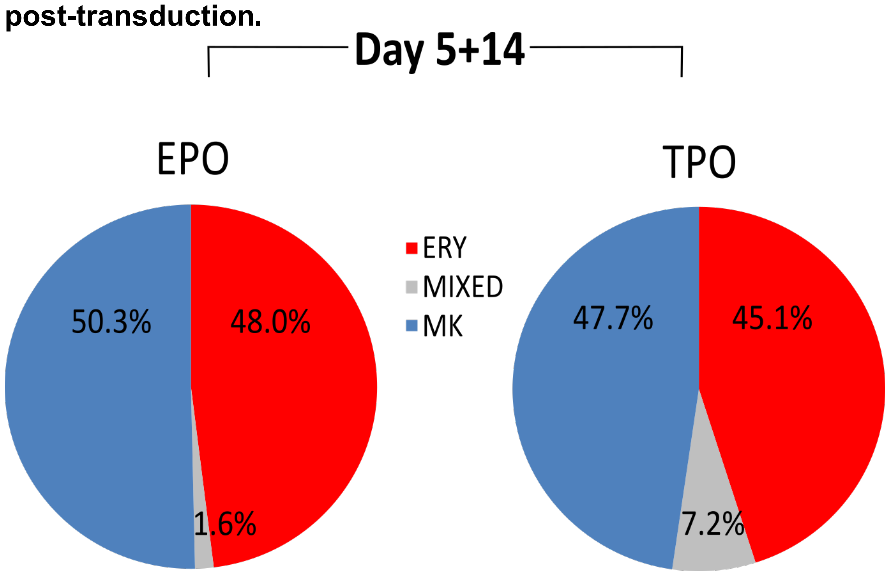

Supplementary Figure 4. Clonogenic assay with forward programmed cells seeded at day 5 post-transduction. Day 5 forward programmed cells cultured in either EPO (left) or TPO (right) were seeded into clonogenic assays and the colony types recorded after 14 days. Mixed colonies are rare (less than 10%) in both conditions and the number of MK or ERY colonies are equally balanced regardless of the cytokine the cells were grown in prior to seeding.

Supplementary Figure 5. Colony forming potential of day 9 BPPs sorted for GT or GFT transgene expression.

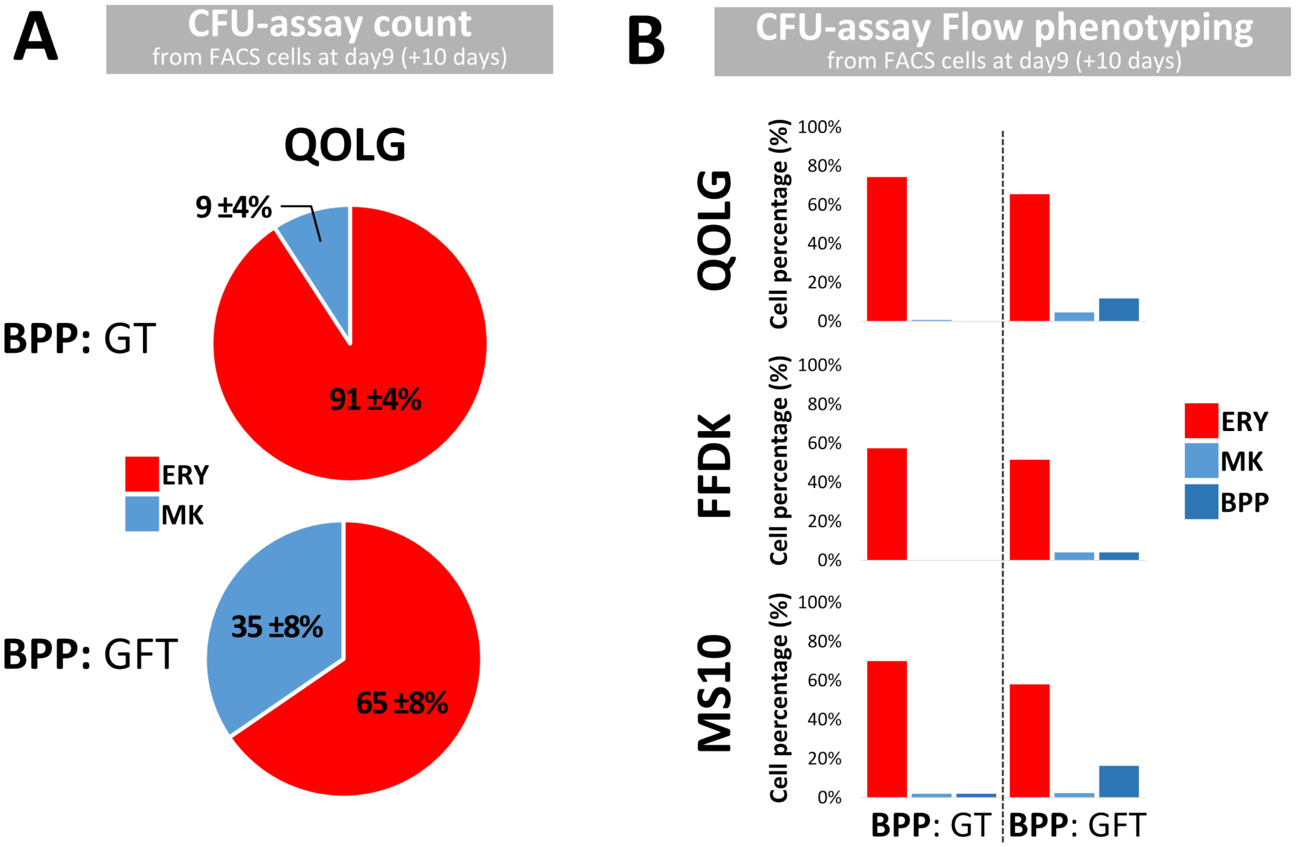

Supplementary Figure 5. Colony forming potential of day 9 BPPs sorted for GT or GFT transgene expression. Additional hPSC lines were forward programmed with rainbow vectors and the BPP population [CD41+/CD235+] was FACS sorted based on its expression of the GATA1+TAL1 [GT] or GATA1+FLI1+TAL1 [GFT] transgenes. The colony potential of each population was assessed in semi-solid methylcellulose clonogenic assays. The number, type (ERY/MK) and phenotype (CD41/CD235a) of colonies were determined after 10 days of culture as per Material & Methods. (**A**) Colony-type distribution after manual counting by 3 different operators of CFU-assay (as per Fig.1E) from biological duplicates of the QOLG cell line (mean +/-SD). (**B**) Phenotyping of the bulk colony assay content by flow cytometry. The total well content was collected and cells stained with CD41 and CD235a antibodies to define the ERY, BPP and MK populations as described in the main text (see Fig.1B). The distribution of the cell populations from 3 hPSC lines are shown (n=1).

Supplementary Figure 6. Expression time course of the haemogenic mesoderm marker CD309/FLK1 in forward programmed cells.

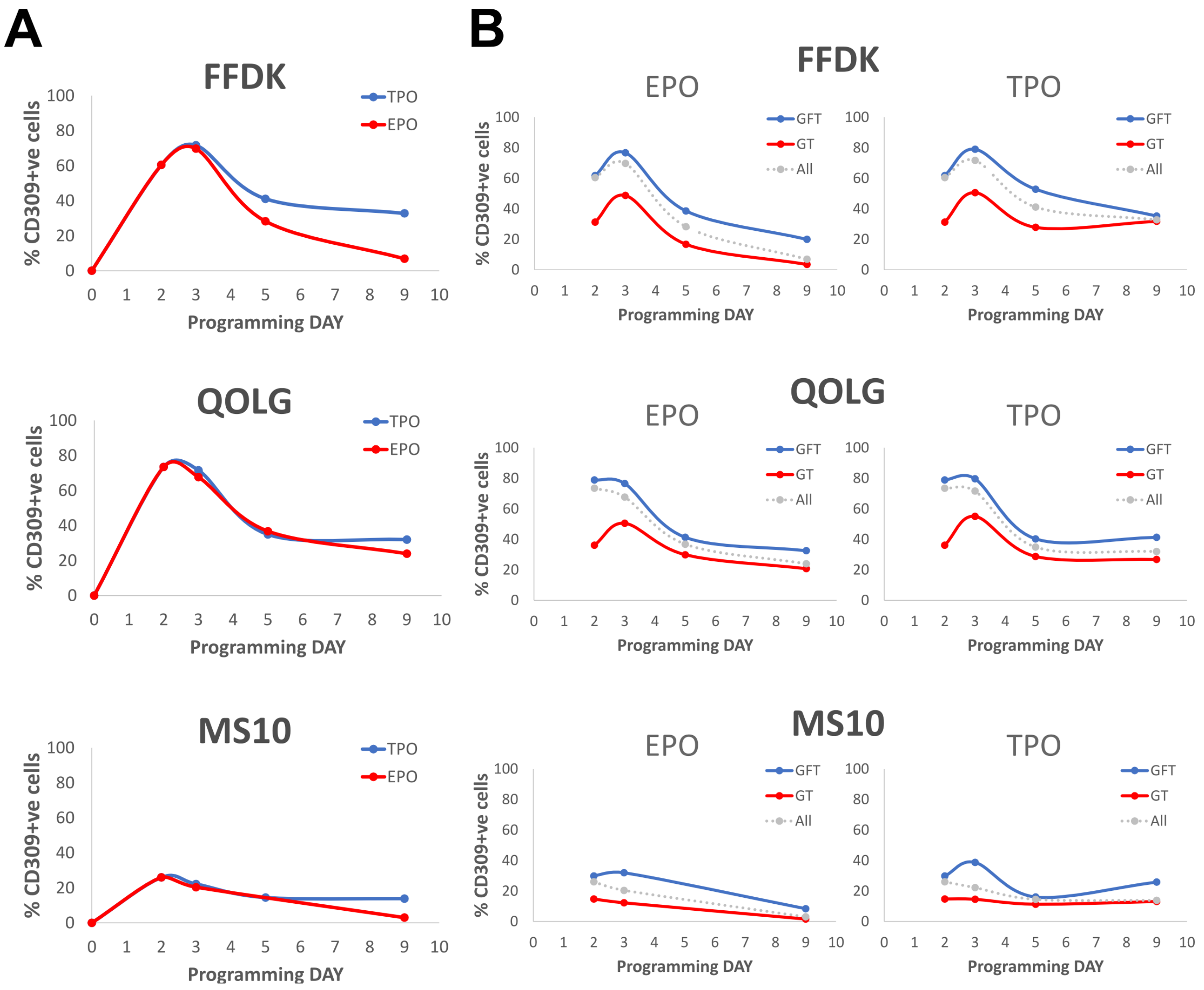

Supplementary Figure 6. Expression time course of the haemogenic mesoderm marker CD309/FLK1 in forward programmed cells. Three hPSC lines (FFDK, QOLG and MS10 lines) were programmed in TPO or EPO conditions with rainbow vectors and were analysed by flow cytometry between day 0-9 for the expression of rainbow transgenes and FLK1-APC. (**A**) The percentage of FLK1+ cells over time is shown for EPO (red) and TPO (blue) cultures for the 3 lines. (**B**) The percentage of FLK1-expressing cells was further analysed specifically inside the [GFT] and [GT] rainbow populations (dark and light blue respectively), vs. the whole live cell gate (grey dotted line).

Supplementary Experimental Procedure

Human pluripotent stem cell culture

The induced hPSC lines A1ATD1c (Yusa et al., 2011),FFDK_1, QOLG_3 (Kilpinen et al., 2017) and embryonic hPSC line MasterSheff-10 (UKSCB) (passage 30 to 50) were cultivated as clumps on feeder-free vitronectin coated wells (VTN-N; ThermoFisher) in a serum-free chemically defined medium (CDM) supplemented with recombinant zebrafish FGF2 and human Activin-A (15 ng ml^{-1} each; Cambridge Stem Cell Institute). The CDM was based on *Chen et al.* (Chen et al., 2011) and prepared with DMEM/F12-HEPES supplemented by 0.054% $NaHCO_3$, 20mg/L insulin, 11mg/L transferrin, 0.0134mg/L selenium (ThermoFisher) and 64mg/L L-Ascorbic Acid (Sigma Aldrich). All three iPSC lines have been derived from adult dermal fibroblasts using integrative murine retroviral vectors (A1ATD1c), episomal DNA vectors (FFDK_1) or non-integrative Sendai vectors (QOGL_3) for expression of the human OCT4, SOX2, KLF4 and MYC reprogramming factors. All cell cultures were maintained at 37°C and 5%CO_2 in humidified incubators.

Recombinant lentiviral vectors

The recombinant lentiviral vectors for expression of the three FoP factors have been previously described (Moreau et al., 2016) and are available at Addgene (ID.92415-7). Briefly, the coding sequences of variant-1 isoforms of GATA1, FLI1 and TAL1 (NM_002049.3, NM_002017.4 and NM_003189.5 NCBI Refseq, respectively) were inserted in place of eGFP into the 2nd generation replication deficient self-inactivating pWPT lentiviral backbone from the Trono lab (Addgene, ID.12255), driving ubiquitous transgene expression from the human EF1α promoter. To generate the rainbow vectors, reporter gene sequences (eGFP, dTomato and LSSmOrange) minus their stop-codon followed by the E2A self-cleaving peptide sequence were generated by PCR from plasmid templates. The obtained reporter-E2A fragments were individually cloned into the pWPT backbone replacing the eGFP. The three FoP factor-coding sequences were subsequently cloned in frame downstream the E2A using bespoke SalI/PspXI restriction sites. All recombinant constructs were sequence-verified.

VSV-G pseudotyped amphotropic lentiviral particles were produced by transient co-transfection of the recombinant vectors with the 2nd generation packaging plasmids pMD2.G and psPAX2 (Addgene, ID.12259-60, respectively) and concentrated 100-fold by PEG precipitation (Takara). Functional titres were determined by qPCR measurement of provirus copy number in genomic DNA or by flow cytometry detection of reporter gene in transduced HCT116 cells (ATCC CCL-247). Alternatively, lentiviral particles were produced commercially to >1E+9TU/mL as 3-vector mix (Vectalys). Transduction of hiPSC lines was performed by single exposure for 16-24 hours in a protamine sulphate-containing medium (10μg/mL) with multiplicity of infection (MOI) of 20 and 80 for each FoP vector and rainbow vector mix, respectively, leading to >80% transduction efficiencies.

Forward Programming

24 hours prior to transduction (day -1), sub-confluent (50–80%) hiPSC cultures were dissociated to small clumps using 0.5 mM EDTA DPBS, before seeding approximately 1E+5 cells onto one well of a vitronectin-coated 12 well plates. On transduction day (day 0), lentiviral particles were added to mesoderm medium -CDM supplemented with FGF2 (20 ng/mL), LY-294002 (10 µM, Sigma), BMP4 (10 ng/mL, Biotechne) and protamine sulphate (10 μg/mL). After 24 hours (day 1), cells were washed and fresh mesoderm media added without protamine sulphate. 24 hours later (day 2), culture media was changed to either erythroblast (ERY) or megakaryocyte (MK) medium, with half-volume renewal every 3 days. The ERY medium was composed of CellGro SCGM (CellGenix), EPO (2 U/mL, Biotechne), SCF (50 ng/mL, ThermoFisher), IL3 (10 ng/mL, Biotechne), insulin (20 µg/mL, Roche) and transferrin (60 µg/mL, Roche). The MK medium, CellGro SCGM, was supplemented with TPO (20 ng/mL, Biotechne) and SCF (25 ng/mL). At day 9 post-transduction, floating cells were collected from the supernatant and pooled with the adherent fraction of the culture dissociated to single cells using TrypLE (ThermoFisher). The single cell preparation was further cultivated in suspension at 2E+5 cells/mL on standard tissue culture vessels (Corning) for an additional 11 days in ERY or MK

medium as described above, including half medium renewal every 3 days and 1:4 cell split when cultures reached >1E+6 cells/mL.

Cord-blood Erythroblast Culture
CD34+ haematopoietic progenitors were isolated from human cord-blood samples obtained as part of an ethically approved study with informed consent by magnetic beads separation according to the manufacturer's protocol (Miltenyi Biotech Ltd). The cells were subsequently cultured in a three-stage protocol as previously described (Griffiths et al., 2012).

Flow cytometry analysis
Day 9 single-cell suspensions generated as above or day 20 cells directly collected from suspension cultures (up to 2E+5 cells) were stained for 20 min at room temperature in 50µL flow buffer (DPBS, 0.5% BSA, 2mM EDTA) using combinations of FITC, PE, PE-Cy7, APC and APC-H7 conjugated antibodies (Table S1), then washed with flow buffer once before analysis. Background fluorescences were set against fluorochrome-matched isotype control antibodies and compensation matrices defined using single-colour-stained cells. The rainbow reporter proteins were used in combination with PE-Cy7, APC and APC-H7 conjugated antibodies only, using single-reporter transduced cells for compensation calculation (Supplementary Figure 5). Flow count fluorospheres (Beckman Coulter) and DAPI (1µg/mL; Sigma Aldrich) were used to determine absolute viable cell count in samples. Enucleation was assessed by DRAQ5 (Biolegend) stain following manufacturer's instructions. Cells were co-stained with an anti-CD235a antibody to determine the percentage of CD235+ cells without nucleus. Flow cytometry experiments were performed on a Gallios flow cytometer and analysed using Kaluza 1.5a software (Beckman Coulter).

Colony forming unit assays
The clonogenic potential of FoP cultures was assessed in semi-solid methylcellulose medium supplemented with serum, EPO, SCF, IL-3, IL-6, G-CSF and GM-CSF (Stemcell Technologies, H4435). After flow cytometry analysis, single cell suspensions were seeded in 1.1mL containing 3.3E+3 cells plated in duplicate in high humidity chambers. The quantitative and qualitative colony outcome was monitored after 14 days upon blind microscopic observation from at least 2 people and results averaged. Flow cytometry analyses of colony assays were performed as above after picking individual colonies or dilution and collection of the whole culture wells in DPBS.

Gene expression analysis by RT-qPCR
Total RNA was extracted from pelleted cultured cells using RNeasy kits (Qiagen) according to the manufacturer's instructions and including DNase treatment. cDNA was prepared from 250–500 ng total RNA using Superscript III Reverse Transcriptase (ThermoFisher) according to the manufacturer's instructions and using random hexamers for priming. qPCR reactions were performed in duplicate using SYBR green chemistry on the ABI 7500HT or Mx3000P instruments (Applied Biosystems; Agilent Technologies). The relative gene expression was calculated by the $2^{-\Delta ct}$ method using GAPDH as housekeeping gene for normalisation. RT-qPCR primer pairs (Table S2) were designed to amplify only cDNA, detect all known isoforms and have no reported off-target matches against the human NCBI RefSeq database. All primers were tested before use for high PCR efficiencies and single dissociation curves. We used UTR priming (absent from transgene sequences) to specifically monitor endogene expression while transgene expression was selectively measured using a common reverse primer specific to the viral vector sequence.

Single cell RNA sequencing of rainbow sorted cells
Single cell library preparation. A forward programmed culture (TPO condition; day40: >99% CD41+, 76% CD42+) was single cell sorted by flow cytometry and 24 single cells from each of the 8 possible transgenic populations (from combination of the 3TFs) collected. The single cells were sorted in 96-well plates containing cell lysis buffer (0.2% Triton X-100 with RNase inhibitor) and frozen to -80°C before library preparation. The method for cDNA library making has been adapted from Smartseq2

for full-length RNAseq from single cells (Picelli et al. 2014). Briefly, the reverse transcription was initiated with oligo-dT primers using SuperScript-II reverse transcriptase and supplemented with ERCC RNA Spike-In (ERCC92, Thermo Fisher). The cDNA was then PCR amplified for 21 cycles using the KAPA HiFi Hotstart ReadyMix and IS primers (Kappa biosystems). The Nextera XT DNA chemistry (Illumina) was used to generate 96-cell indexed libraries. The sequencing was performed on an HiSeq-4000 system (Illumina; one 96-cell library per lane).

Single cell sequencing analysis. Raw sequencing reads (fastq files) were aligned to the human Ensembl reference genome GrCh38.81 supplemented with sequences for ERCC spike-in genes and the three fluorescent reporters eGFP, IssmOrange and dTomato expressed exogenously in FoP-MKs. Alignment was performed with GSNAP (Wu et al. 2010) with parameters *-n 1 -Q,* to discard multi-mapping reads. Unstranded uniquely mapped reads were then assigned to genes with HTSEQ-Count (Anders et al. 2015) with parameter *-s no*. Data was then quality-checked by discarding all cells with less than 400,000 read counts or showing high proportion of mitochondrial RNA reads (>10%) or ERCC spike-in reads (>2%), leaving 111/196 cells for further analyses. All non-detected genes (read count equal to zero for all cells) were removed. Read counts were then normalised with the scran algorithm (Lun et al., 2016) with parameters *min.sizes=20* and *sizes=10*. The sequences have been deposited with the following GEO accession number: GSE121579

Cell morphology

1-2E+5 single cells in suspension were spun onto a glass slide using cytofunnels at 400*g* for 5 minutes, methanol fixed, stained using the Giemsa (VWR) staining method and observed under phase contrast microscope.

Western blotting

Cells were lysed in RIPA buffer containing proteinase inhibitors (complete, Roche) and sonicated for 5 minutes. Samples were resolved by SDS-PAGE, transferred to a PVDF membrane (Millipore), blocked with 10% milk and incubated for 1 hour in specific antibodies for α, β, γ and ξ-globin and β-actin characterisation (table S1). Membranes were then incubated in specific HRP-conjugated secondary antibodies for 1 hour and bands were visualized using enhanced chemilunescence (G.E. Healthcare).

Statistics

Regression analysis.

The data for the Colony Forming Unit (CFU) experiments were analysed using mixed-effects Poisson regression in which the outcome was the number of colonies formed, after pooling counts within experimental duplicate. The model included a random effect corresponding to each technical operator and fixed effects representing replicate number, condition (i.e., cytokine for the first experiment and transcription factor combination for the second experiment) and an interaction between condition and cell type.

The flow cytometry data were analysed using mixed effects logistic regression. The binary outcome indicated whether a cell was CD41+CD235- (MK) or CD41+CD235+ (BPP) in one analysis and CD41-CD235+ (ERY) or CD41+CD235+ (BPP) in another analysis. The models included a fixed effect for each replicate and random slopes with common variance representing the replicate-specific changes in the log odds due to cytokine exposure (e.g. TPO vs EPO). The random slopes accounted for evident variation in the treatment effects across replicates (Supplementary Figures 3A-C).

The models for CFU and flow cytometry experiments were fitted using the glmer() function from the lme4 R package. Nested models fitted with the glmer() function were compared using the anova() R function.

General analysis.

Results are presented as mean ± SEM or SD, with *n* representing the number of biological replicates. P-values derived from applying either two-tailed Student's *t*-tests of a difference in means between

two samples or one-way ANOVA Tukey post-hoc test. These are mentioned in figure legends, where applicable. Statistical significance are indicated in the figures as follows: * for P-value<0.05, ** for P-value<0.01 and *** for P-value<0.001.

Vector maps

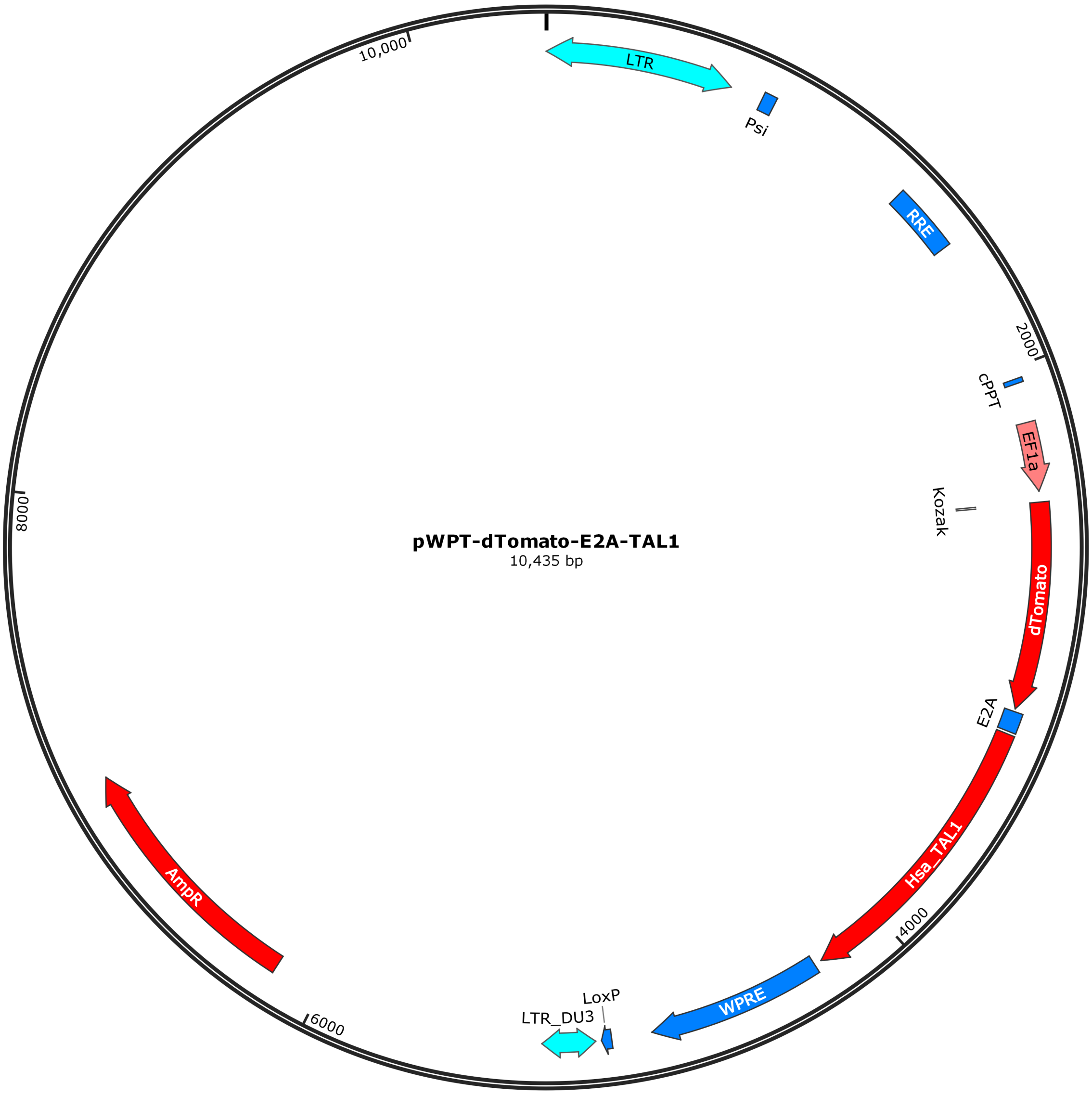

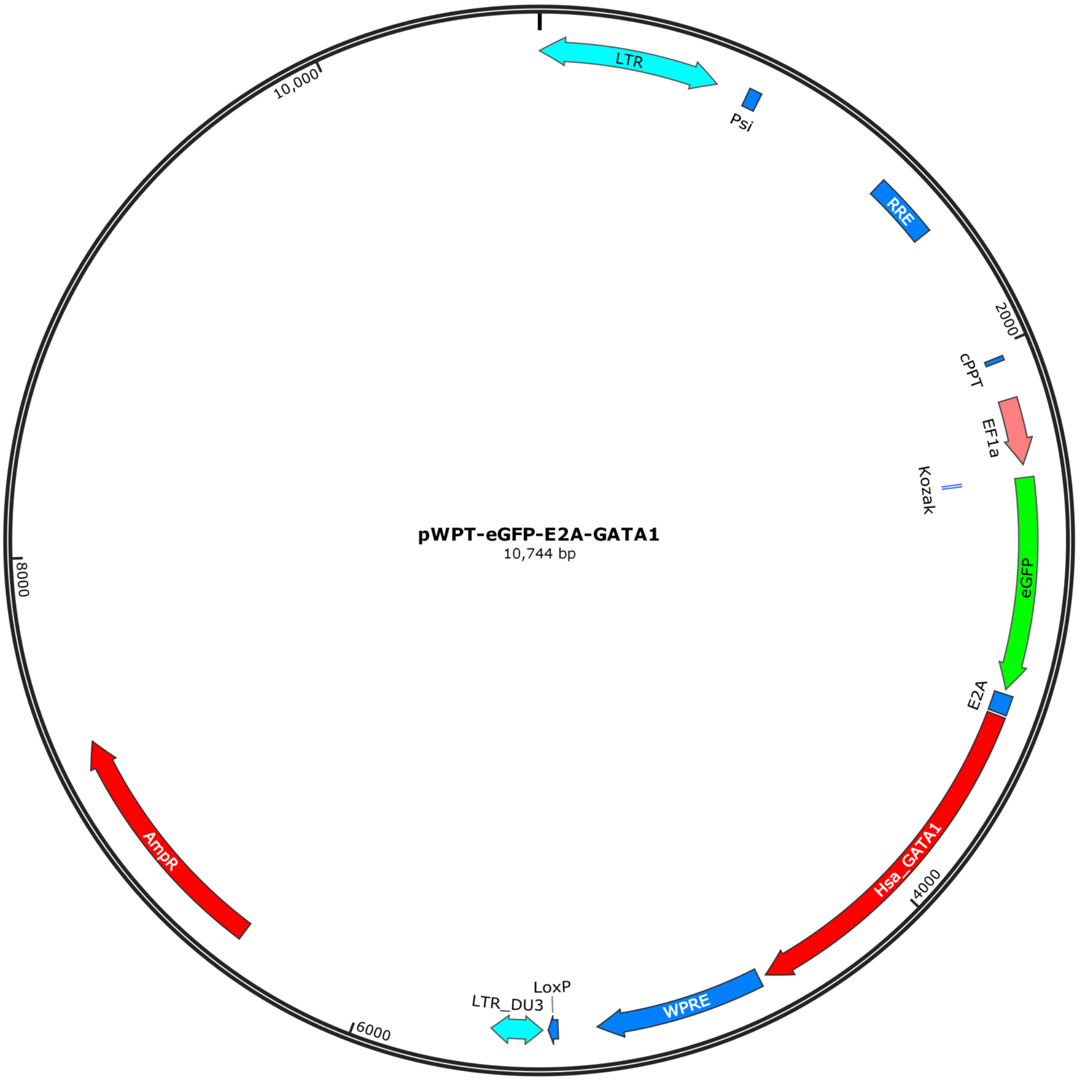
pWPT-eGFP-E2A-GATA1
10,744 bp
LTR
Psi
RRE
2000
cPPT
EF1a
Kozak
eGFP
E2A
Hsa_GATA1
4000
WPRE
LoxP
LTR_DU3
6000
AmpR
8000
10,000

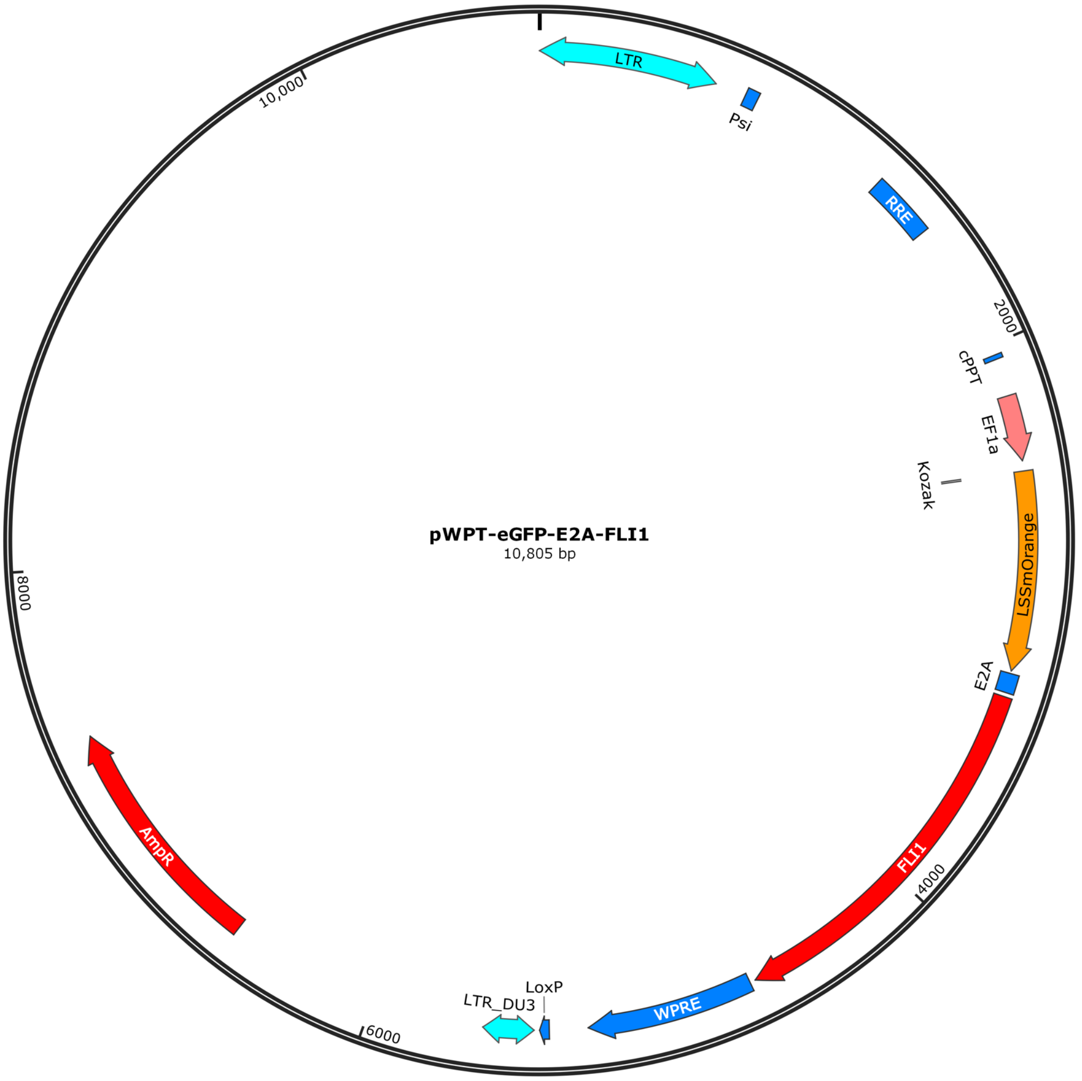

pWPT-eGFP-E2A-FLI1
10,805 bp
LTR
Psi
RRE
2000
cPPT
EF1a
Kozak
LSSmOrange
E2A
FLI1
4000
WPRE
LoxP
LTR_DU3
6000
AmpR
8000
10,000

Table S1. Antibodies used for flow cytometry analyses and western blotting.

Antigen	Antibody/Clone	Fluorochrome	Manufacturer
CD14	MφP9	FITC	BD Pharmingen
CD41a	559777	APC	BD Pharmingen
CD42a	558818	FITC	BD Pharmingen
CD66c	551478	PE	BD Pharmingen
CD71	M-A712	PE	BD Pharmingen
CD235a	555570	PE	BD Pharmingen
CD235a	GA-R2 (HIR2)	PE-Cy7	BD Pharmingen
BAND3	BRIC6-PE	PE	IBGRL Research Products
RhD	BRAD3-PE	PE	IBGRL Research Products
α-globin	sc-514378		Santa Cruz
β -globin	sc-21757		Santa Cruz
γ-globin	sc-21756		Santa Cruz
ξ-globin	ab156041		Abcam
β-actin	A5441		Sigma

Table S2. Oligonucleotides used in the study for qPCR analysis.

Target gene	Primer	Sequence (5'-3')
GATA1 (all)	Forward	CAGAACAGGCCCCTCATCC
GATA1 (all)	Reverse	TCAGTGGCCGGTTCACCT
GATA1 (TG)	Forward	GGTGGCTCCGCTCAGCTCAT
GATA1 (TG)	Reverse	GCAGCGTATCCACATAGCGTAAAAGG
GATA1 (endo)	Forward	TTGCCACATCCCCAAGGCGG
GATA1 (endo)	Reverse	GGGGGAGGGGCTCTGAGGTC
FLI1 (all)	Forward	ATACAACCTCCCACACCGAC
FLI1 (all)	Reverse	CTGATACGGATCTGGCTGGG
FLI1 (TG)	Forward	CCCGCCATCCTAACACCCACG
FLI1 (TG)	Reverse	GCAGCGTATCCACATAGCGTAAAAGG
FLI1 (endo)	Forward	GGGCTCGGCTGCAGACTTGG
FLI1 (endo)	Reverse	AGATGGGCTGCCGCTCCGTA
TAL1 (all)	Forward	GGAGACCTTCCCCCTATGAGA
TAL1 (all)	Reverse	CCCGGCTGTTGGTGAAGATA
TAL1 (TG)	Forward	AGGCGGTGGACTTGAACTTT
TAL1 (TG)	Reverse	TCTAGCCAGGCACAATCAGC
TAL1 (endo)	Forward	AGCAAAGACCCGGGTGTGCATC
TAL1 (endo)	Reverse	CCTCTAGCTGGGGGTCACTGCG
GAPDH	Forward	TATCGTGGAAGGACTCATGACC
GAPDH	Reverse	TAGAGGCAGGGATGATGTTCTG
KLF1	Forward	TGACTTCCTCAAGTGGTGGC
KLF1	Reverse	AGTTGGTGAGGAGGAGATCCA
HBB	Forward	TGGCAAGAAAGTGCTCGGTG
HBB	Reverse	CAGCTCACTCAGTGTGGCAAAG
HBE1	Forward	GGGCAGACTCCTCGTTGTTT
HBE1	Reverse	CCACATGCAGCTTGTCACAG
HBG	Forward	ATGGCAAGAAGGTGCTGACT
HBG	Reverse	TGGATTGCCAAAACGGTCAC
HBA	Forward	CCGACAAGACCAACGTCAAG
HBA	Reverse	GTCGAAGTGCGGGAAGTAGG
