# Supplementary material for: Transcription Factor Levels after Forward Programming of Human Pluripotent Stem Cells with GATA1, FLI1, and TAL1 Determine Megakaryocyte versus Erythroid Cell Fate Decision
Source: Stem Cell Reports. 2018 Nov 29;11(6):1462–78. doi: 10.1016/j.stemcr.2018.11.001 (PMC6294717; doi:10.1016/j.stemcr.2018.11.001)
Supplement: Document S2. Article plus Supplemental Information [file mmc2.pdf]

Stem Cell Reports
Article
OPEN ACCESS

Transcription Factor Levels after Forward Programming of Human Pluripotent Stem Cells with GATA1, FLI1, and TAL1 Determine Megakaryocyte versus Erythroid Cell Fate Decision

Amanda Dalby,[1,2,3] Jose Ballester-Beltrán,[1,2,3] Chiara Lincetto,[1,2] Annett Mueller,[1,2] Nicola Foad,[1,2] Amanda Evans,[1,2] James Baye,[2] Ernest Turro,[1] Thomas Moreau,[1,2,4] Marloes R. Tijssen,[1,2,4] and Cedric Ghevaert[1,2,4,*]

[1]Department of Haematology, University of Cambridge and NHS Blood and Transplant, Cambridge Blood Centre, Long Road, Cambridge CB2 0PT, UK
[2]Wellcome Trust-Medical Research Council Cambridge Stem Cell Institute, Tennis Court Road, Cambridge CB2 1QR, UK
[3]Co-first author
[4]Co-senior author
*Correspondence: cg348@cam.ac.uk
https://doi.org/10.1016/j.stemcr.2018.11.001

SUMMARY

The production of blood cells and their precursors from human pluripotent stem cells (hPSCs) *in vitro* has the potential to make a significant impact upon healthcare provision. We demonstrate that the forward programming of hPSCs through overexpression of GATA1, FLI1, and TAL1 leads to the production of a population of progenitors that can differentiate into megakaryocyte or erythroblasts. Using "rainbow" lentiviral vectors to quantify individual transgene expression in single cells, we demonstrate that the cell fate decision toward an erythroblast or megakaryocyte is dictated by the level of FLI1 expression and is independent of culture conditions. Early FLI1 expression is critical to confer proliferative potential to programmed cells while its subsequent silencing or maintenance dictates an erythroid or megakaryocytic fate, respectively. These committed progenitors subsequently expand and mature into megakaryocytes or erythroblasts in response to thrombopoietin or erythropoietin. Our results reveal molecular mechanisms underlying hPSC forward programming and novel opportunities for application to transfusion medicine.

INTRODUCTION

Blood transfusion is a common medical procedure used for a large number of pathologies. Cancer treatment may cause bone marrow failure (myelosuppression) and result in a decreased platelet and red blood cell count in the peripheral blood (thrombocytopenia and anemia, respectively). Blood loss, post-trauma or following surgery, can lead to similar issues. Thrombocytopenia results in a high risk of bleeding. Anemia leads to deficient oxygen transport to organs (hypoxemia). Currently, provision of blood products for transfusion relies on blood donations, which has risen to 112 million annual collections globally. Demographic projections expect an overall drop in the number of people within the age group allowed to donate blood by 2050, as well as an increase in population >60 years of age needing blood transfusion treatments (Ali et al., 2010). This trend is compounded by an increase in the use of aggressive cancer therapies and of more advanced surgical procedures, necessitating more blood transfusions. The reliance on allogeneic blood products has logistic and financial implications, along with other challenges such as maintaining biosafety and immunological compatibility between donor and recipient. Considerable efforts to overcome this dependency have been made during the last few decades, which include the *ex vivo* production of blood cells (reviewed in Palmer and Intaglietta, 2014).

The *ex vivo* production of blood cells has focused first on the differentiation of multi-potent stem/progenitor cells, such as CD34+ cells extracted from cord or peripheral blood, and more recently on the use of human pluripotent stem cells (hPSCs), such as embryonic stem cells (ESCs) (Hirose et al., 2013) or induced pluripotent stem cells (iPSCs) (Kurita et al., 2013). Human PSCs can be maintained *in vitro* for prolonged periods while retaining the capacity to differentiate toward virtually any cell type upon adequate stimulation. Therefore, they represent a huge opportunity as a source of renewable stem cells from which clinically relevant cellular therapies can be derived (Leach and Clegg, 2015; Lilly et al., 2016; Stoker and Barker, 2016). hPSC differentiation protocols have usually delivered sequential environmental signals to the cells through the use of culture media and different cytokine cocktails through a multi-stage differentiation process, mimicking developmental pathways (Irion et al., 2008). These "directed differentiation" protocols may deliver relatively low numbers of cells at suboptimal purity and are commonly complex and come at a significant cost.

We have recently described a novel approach for generating megakaryocytes (MKs) from hPSCs using a forward-programming (FoP) approach (Moreau et al., 2016). FoP relies on the fact that cell identity is controlled by a given set of transcription factors (TFs), and that the exogenous expression of a specific master TF subset governing the

This is an open access article under the CC BY license (http://creativecommons.org/licenses/by/4.0/).

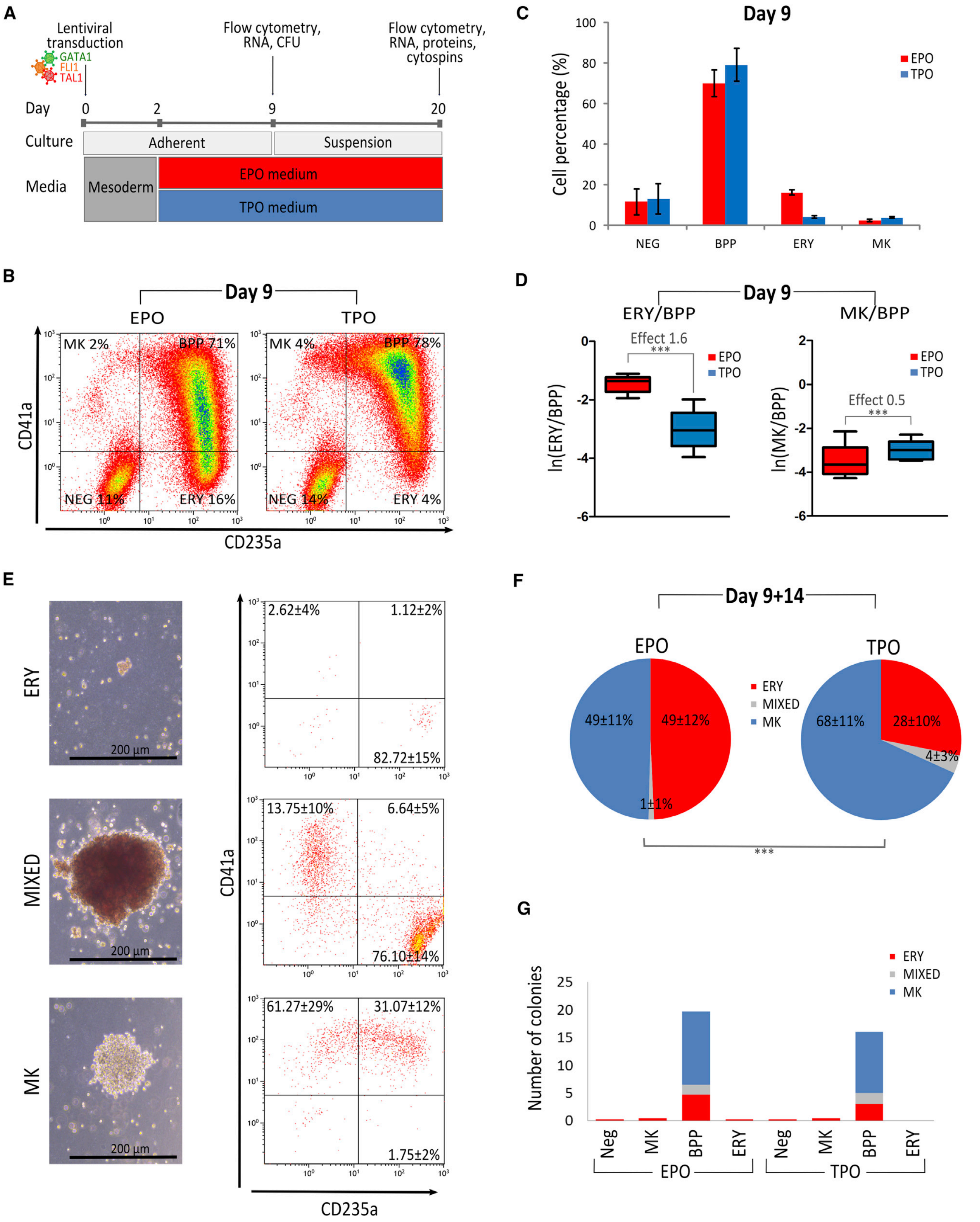

(legend on next page)

entire gene regulatory network can enforce cellular identity. We showed that FoP, by the overexpression of GATA1, FLI1, and TAL1 in hPSCs, leads to the production of a large quantity of highly pure MKs from which functional platelets can be harvested.

Two of the FoP TFs used to produce MKs, namely GATA1 and TAL1, are well known to have crucial roles in the differentiation of the erythroid lineage (Han et al., 2016), and it is well documented that MKs and erythroblasts (ERYs) are developmentally closely related through the hematopoietic ontogeny (reviewed in Dore and Crispino, 2011).

In this paper we show that, in fact, FoP of hPSCs with GATA1, FLI1, and TAL1 leads to the generation of a bipotent population (BPP) in the early stages of culture from which both MKs and erythroid cells can be derived. We demonstrate that the bifurcation toward one lineage or the other is primarily dictated by FLI1 transgene expression levels, while lineage-specific cytokines (such as thrombopoietin [TPO] and erythropoietin [EPO]) enable the expansion and differentiation of the cells already committed toward the MK or ERY lineage, respectively.

RESULTS

Overexpression of GATA1, FLI1, and TAL1 in hPSCs Generates a Population of Progenitors with Megakaryocytic and Erythroid Differentiation Potential

GATA1, FLI1, and TAL1 (hereafter 3TFs) were overexpressed concurrently by transducing hPSCs with individual lentiviral vectors expressing each of the 3TFs. After 2 days in a medium promoting mesoderm differentiation, the culture condition was changed to either an ERY or MK differentiation medium containing EPO or TPO, respectively (Figure 1A).

Cells were harvested at day 9 post-transduction, and the surface expression of CD235a (glycophorin A; an erythroid marker) and CD41a (integrin αIIb, ITGA2B; an MK lineage marker) was measured by flow cytometry. We then defined three cell populations based on these two phenotypic markers: the BPP (CD41+/CD235+ cells), MK (CD41+/CD235−), and erythroid (ERY) (CD41−/CD235+) populations. In both the EPO and TPO culture conditions the majority of cells belonged to the BPP (70% ± 7% and 79% ± 8% in the EPO and TPO condition, respectively, Figures 1B and 1C). Untransduced hPSCs cultured in the same media did not acquire either of these markers (Figure S3A). The number of viable cells and the proportion of BPPs found in the EPO versus the TPO culture condition were not statistically significantly different, but the probability of the remaining cells being ERY or MK rather than BPP were significantly associated with the cytokine used ($p < 0.001$) in the culture medium. We estimated the change in the log odds of cells being of a particular type rather than another (hereafter, “mean effect”) using a mixed effect model with random slope to account for experimental variations (see Figure S1A). The mean effect is calculated across biological replicates. We found that, at day 9 post-transduction, EPO had a positive effect on ERY production (mean effect 1.6), and that was also the case for TPO on MK production, albeit less pronounced (mean effect 0.5) (Figure 1D).

To study the differentiation potential of the BPP, cells were harvested in bulk at day 9 post-transduction and seeded into clonogenic assays in semi-solid medium containing a range of hematopoietic cytokines supporting pan-myeloid differentiation (confirmed using cord blood [CB]-derived CD34+ hematopoietic progenitor cells [HPCs]). First of all, FoP cells harvested from both EPO and TPO culture conditions never produced any granulocytic or monocytic colonies, confirming the restriction of

Figure 1. Forward Programming of hPSCs with GATA1, FLI1, and TAL1 Generates a Bipotent Megakaryocyte/Erythroid Population

(A) Schematic representation of the experimental setup. Following transduction with three lentiviruses and an initial mesoderm-promoting 2-day culture, cells were divided into an erythroid (EPO) or MK (TPO) culture.

(B and C) Cells from the EPO and TPO culture conditions were analyzed by flow cytometry at day 9 post-transduction. (B) Representative dot plots for CD41a and CD235a expression and (C) chart summarizing the percentages of cells belonging to the non-programmed (NEG) (CD41a−/CD235a−), bipotent (BPP) (CD41a+/CD235a+), erythrocyte (ERY) (CD41a−/CD235a+), and megakaryocyte (MK) (CD41a+/CD235a−) populations, respectively (n = 5, mean ± SEM); EPO culture (red bars), TPO culture (blue bars).

(D) Boxplots over five replicates of the log(e) of the number of ERYs (left plot) and MKs (right plot) divided by the number of BPPs as detected by flow cytometry at day 9. The log odds of cells being ERY rather than BPP (“mean effect”) in EPO (versus TPO) was 1.6, while the log odds of a cell being an MK rather than BPP in TPO (versus EPO) was 0.5 (***$p < 0.001$).

(E and F) The colony potential of day 9 cells was assessed by colony-forming unit (CFU) assays. Cells were cultivated in duplicate in semi-solid medium and the colony outcome (number and morphology) analyzed blindly by at least two operators after 14 days. (E) Exemplars of the three types of colonies recorded, including erythroid (ERY), megakaryocytic (MK), or mixed colonies containing both erythroid and MK cell types (MIXED). (F) The pie charts show CFU distribution depending on culture condition (EPO versus TPO; n = 4, mean% ± SEM); ERY in red, MK in blue and MIXED in gray. Poisson regression analysis shows that the distribution of the number of colonies per cell type depends on cytokine exposure (***$p < 0.001$).

(G) Colony potential of day 9 cells sorted according to their cell surface markers. Only cells expressing both CD235a and CD41a showed colony-forming potential.

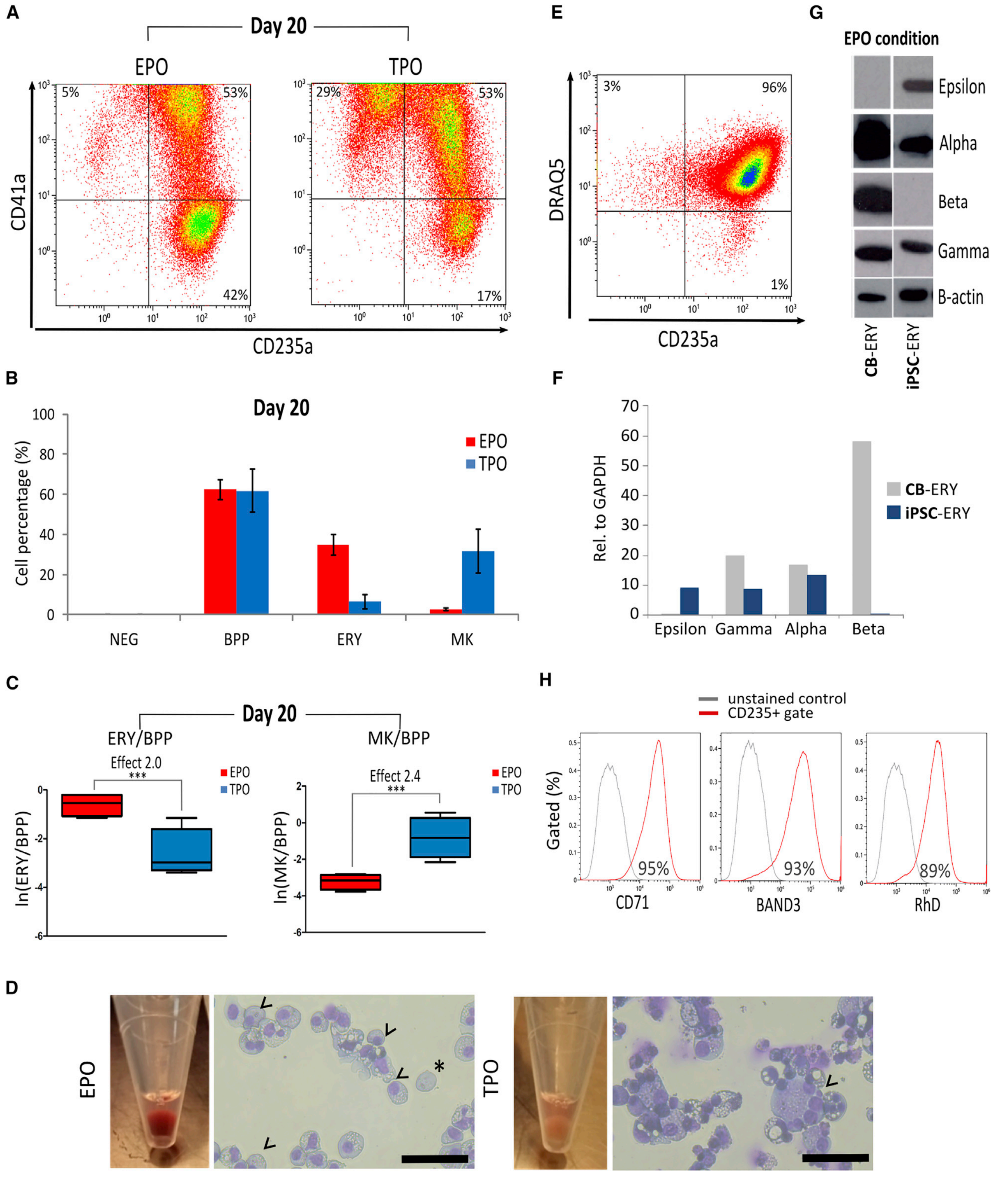

Figure 2. Maturation of the Forward Programmed hPSCs

Day 9 FoP cells were further cultured in suspension in EPO or TPO conditions and cell differentiation analyzed at day 20 post-transduction.
(A) Representative flow cytometry dot plots for CD41a and CD235a expression.

(legend continued on next page)

their differentiation potential to the MK and ERY lineages (Figure S2).

After 14 days, colonies were counted and subdivided into three morphological categories: ERY (small red colonies), MK (large white colonies), and MIXED (large red colonies). Representative micrographs of the different colony subtypes are shown in Figure 1E. Flow cytometry analysis of individually picked colonies confirmed the morphological classification: ERY colonies contained predominantly CD235a+ CD41− cells, MK colonies contained cells that were all CD41a+, while MIXED colonies contained combinations of cells that were either CD235a+ CD41a− or CD41a+ CD235a− (Figure 1E). Day 9 cells harvested from either the EPO or TPO condition were able to form colonies of all three subtypes, confirming the dual lineage potential of the early culture as a whole. MIXED colonies were rare in both conditions, representing single-figure percentage of all colonies (Figure 1F). However, the distribution of the other two colony types differed significantly ($p < 2 \times 10^{-16}$) between the two conditions. In the EPO condition, approximately the same numbers of ERY and MK colonies were produced, whereas the cells grown under the TPO condition produced over twice as many MK than ERY colonies. We sorted day 9 FoP cells according to expression of CD41a and CD235a into four populations (non-programmed, ERY, MK, and BPP) and demonstrated that the colony-forming potential was unique to the BPP, thereby confirming that the progenitors are contained solely within this double-positive CD41a+ CD235a+ population (Figure 1G).

Forward Programmed Cells Mature into ERYs or MKs when Cultured with Medium Containing EPO or TPO, Respectively

Day 9 FoP cells were cultured for a further 11 days in medium containing either EPO or TPO. By day 20 of culture, the total number of viable cells represented on average a 174-fold expansion per starting hPSC in the EPO condition and 66-fold expansion in TPO (Figure S3C). Flow cytometry at day 20 post-transduction showed that the predominant population of cells in both conditions was still the double-positive BPPs. However, in EPO the percentage of ERYs had significantly increased from day 9, reaching 35% ± 5% while ERYs only represented 7% ± 4% of the cells in TPO. Conversely, the percentage of MKs had increased to 32% ± 11% in TPO while MKs represented 3% ± 1% of the total cells in EPO (mean ± SD, Figures 2A and 2B). The probability of cells being MK rather than BPP in TPO was higher at day 20 (mean effect = 2.4, $p < 0.001$) than it was at day 9 (mean effect = 0.5). Similarly, the probability of cells being ERY rather than BPP in EPO was even higher at day 20 (mean effect = 2.0, $p < 0.001$) than at day 9 (mean effect = 1.6) (Figures 2C and S1B).

Day 20 cell pellets were macroscopically different. A red hemoglobinized cell pellet was seen in the EPO condition, contrasting with a white pellet from the TPO condition (Figure 2D). Giemsa-stained cytospins of the EPO culture showed small cells with a morphology in keeping with pro-erythroblasts and basophilic normoblasts (Figure 2D, left panel). A small fraction of the cells had undergone further differentiation to poly- and ortho-chromatic normoblasts, denoted by their eccentric condensed nuclei and hemoglobinized cytoplasm (Figure 2D, left panel, arrowhead). Terminally differentiated reticulocytes, denoted by the absence of a nucleus, were occasionally seen on cytospins (Figure 2D, left panel, asterisk). Flow cytometry using the nuclear stain DRAQ5 showed that enucleated cells represented a single-figure percentage of the total cells (Figure 2E). The TPO condition supported the growth of megakaryoblasts (Figure 2D, right panel) with occasional polynucleated MKs (Figure 2D, right panel, arrowheads).

(B) Percentages of cells belonging to the undifferentiated NEG (CD41a−/CD235a−), BPP (CD41a+/CD235a+), ERY (CD41a−/CD235a+), and MK (CD41a+/CD235a−) populations, respectively, in the different culture conditions (n = 4, mean ± SEM); EPO culture (red bars), TPO culture (blue bars).
(C) Boxplots over sets of four replicates of the log(e) of the number of ERYs (left plot) and MKs (right plot) over the number of BPPs as detected by flow cytometry. The log odds of cells being ERY or MK (mean effect) rather than BPP were strongly associated with cytokine exposure and were 2.0 (ERY in EPO) and 2.4 (MK in TPO) (***$p < 0.001$).
(D) Pictures of day 20 cell pellets from representative EPO (left) and TPO (right) cultures and cell morphology analyzed by Giemsa staining. Cytospins show normoblasts with condensed eccentric nuclei (arrowheads) and an enucleated reticulocyte (asterisk) from the EPO (left) culture and large polyploid MKs (arrowheads) from the TPO (right) culture. Scale bar, 50 μm.
(E) Representative flow cytometry dot plot of day 20 cells analyzed for expression of CD235a and the nuclear staining DRAQ5. Enucleated reticulocytes (bottom right quadrant) represent only a single-figure percentage of the whole-cell population.
(F) RNA was extracted from day 20 forward-programmed cells (iPSC-ERY) and cord blood-derived erythroblasts (CB-ERY) and analyzed by qRT-PCR to quantify the expression of the ε-, γ-, α-, and β-globin chains (n = 1).
(G) Whole-cell extracts were analyzed for ε-, γ-, α-, and β-globin content by western blotting. The left panels represent the results from cell lysates of CB-ERY and the right panels those from iPSC forward-programmed erythroblasts (iPSC-ERY); β-actin was used as protein loading control (n = 1).
(H) Representative flow cytometry histograms of CD71, BAND3, and RhD expression in CD235a+-gated cells from the EPO cultures; isotype control (gray line) and stained cells (red line).

ERYs derived from hPSCs typically express embryonic and fetal globins (Olivier et al., 2016). Using RNA extracted from day 20 EPO-cultured ERYs, we carried out qRT-PCR and showed expression of the pan-globin α alongside the embryonic (ε) and the fetal (γ) globin chains (Figure 2F). These results were confirmed in western blots, showing substantial expression of the fetal and embryonic globins in the hPSC-derived ERYs, while expression of the adult β globin was only detected in CB HPC-derived ERYs (Figure 2G). We have previously shown that FoP MKs express cell surface proteins typically found in mature MKs, such as the GP1b/V/IX complex GPIIa and GPVI (Moreau et al., 2016), and we confirmed this was the case for the day 20 cells cultured in TPO (data not shown). Conversely, we confirmed that the ERYs produced in the EPO condition showed expression of typical erythroid cell surface markers such as CD71, Band 3, and RhD (Figure 2H).

We went on to select a further 3 hPSC lines (two iPSC lines and one ESC line) for which we had previously documented MK production using 3TFs FoP in TPO. We confirmed that these cell lines forward programmed with the 3TFs and, when cultured in EPO, were equally capable of producing ERYs (Figures S3B–S3D). We noticed that, in this particular set of experiments, the emergence of ERYs versus BPPs happened earlier in the culture as the ERYs already represented the largest portion of cells by day 9 of culture.

Lineage Fate Decision Is Not Dictated by Cytokines

The results described above show that overexpression of the 3TFs in hPSCs leads to the generation of a BPP that is endowed with differentiation potential toward the ERY or MK lineage regardless of whether the cells are cultured in EPO or TPO medium during the first 9 days post-transduction. These BPPs can mature into ERYs or MKs according to the cytokine used beyond day 9.

We set out to understand how the bifurcation to one or the other lineage is regulated, focusing on the particular role played by transgene expression and cytokines.

Two hypotheses were formulated: (1) a cytokine-instructing mechanism where the BPP would be biased toward ERY or MK lineage depending on EPO or TPO treatment or (2) a programming factor-driven lineage commitment where the combination and/or level of expression of the 3TFs primarily dictates the differentiation potential. In this scenario, EPO and TPO would merely provide a survival/proliferative advantage to cells that, although they express both CD235a and CD41a at day 9, are already pre-destined to either the ERY or MK lineage by virtue of their level of transgene expression.

The relatively low number of truly mixed colonies generated by the cells seeded at day 9 (Figure 1F) suggested that the latter hypothesis is correct, and that single cells from the BPP population are already lineage restricted. Furthermore, when cells were harvested at an earlier time point (day 5, the earliest time point when CD235a and CD41a co-expressing cells are present), we confirmed that these cells were already able to form all three types of colonies but, again, MIXED colonies were rare (<7.2%), (Figure S4). This indicates that lineage restriction is a very early event and that truly bipotent progenitor cells represent a very transient step during the programming process.

The colony assays carried out with cells harvested at day 9 showed that ERY- and MK-committed cells co-existed in both EPO and TPO cultures, suggesting that the cytokine used until that point does not restrict lineage commitment. Therefore, the subsequent expansion and maturation beyond day 9 of BPPs that are committed to one lineage early on should not be affected by the cytokine used earlier, between days 2 and 9. We demonstrated this by performing cytokine switch experiments. Cells that were switched from EPO (days 2–9) to TPO (days 9–20) showed an increase in the percentage of MKs compared with cells that were maintained in EPO (17% versus 3%, respectively) (Figures 3A and 3B). Similarly, cells switched from TPO to EPO showed an increase in the percentage of ERYs compared with cells maintained in TPO (31% versus 7%, respectively). The data also revealed that the early cytokine treatment had a much smaller effect on the outcome of the culture compared with the cytokines used between days 9 and 20. Regardless of the initial cytokine used, the mean effect of late TPO treatment on a cell being an MK rather than BPP was 2.2 and 1.6, respectively ($p < 0.001$) while the mean effect of late EPO treatment on a cell being an ERY rather than BPP was 1.9 and 1.6, respectively ($p < 0.001$). By contrast, conditional on the second cytokine, the mean effect of the first cytokine on the MK/BPP and ERY/BPP were not significant in 3 of the 4 comparisons, and had a magnitude of less than 1 in the fourth condition (Figures 3C and S1C).

The fact that lineage restriction is a very early event and that the early cytokine treatment (days 2–9) does not significantly affect the capacity of the BPPs to differentiate subsequently toward the ERY or MK lineage suggests that the 3TFs might play a predominant role over the cytokines in the cell fate decision.

Transgene Expression Dictates Cellular Identity Regardless of the Cytokine Environment

To explore this putative TF-driven lineage switch, we generated "rainbow" vectors to allow the quantification of the expression of each transgenic TF in single cells by flow cytometry. Each TF was cloned into a separate lentiviral vector backbone containing one of three fluorescent proteins: eGFP to report for GATA1, dTomato for TAL1, and

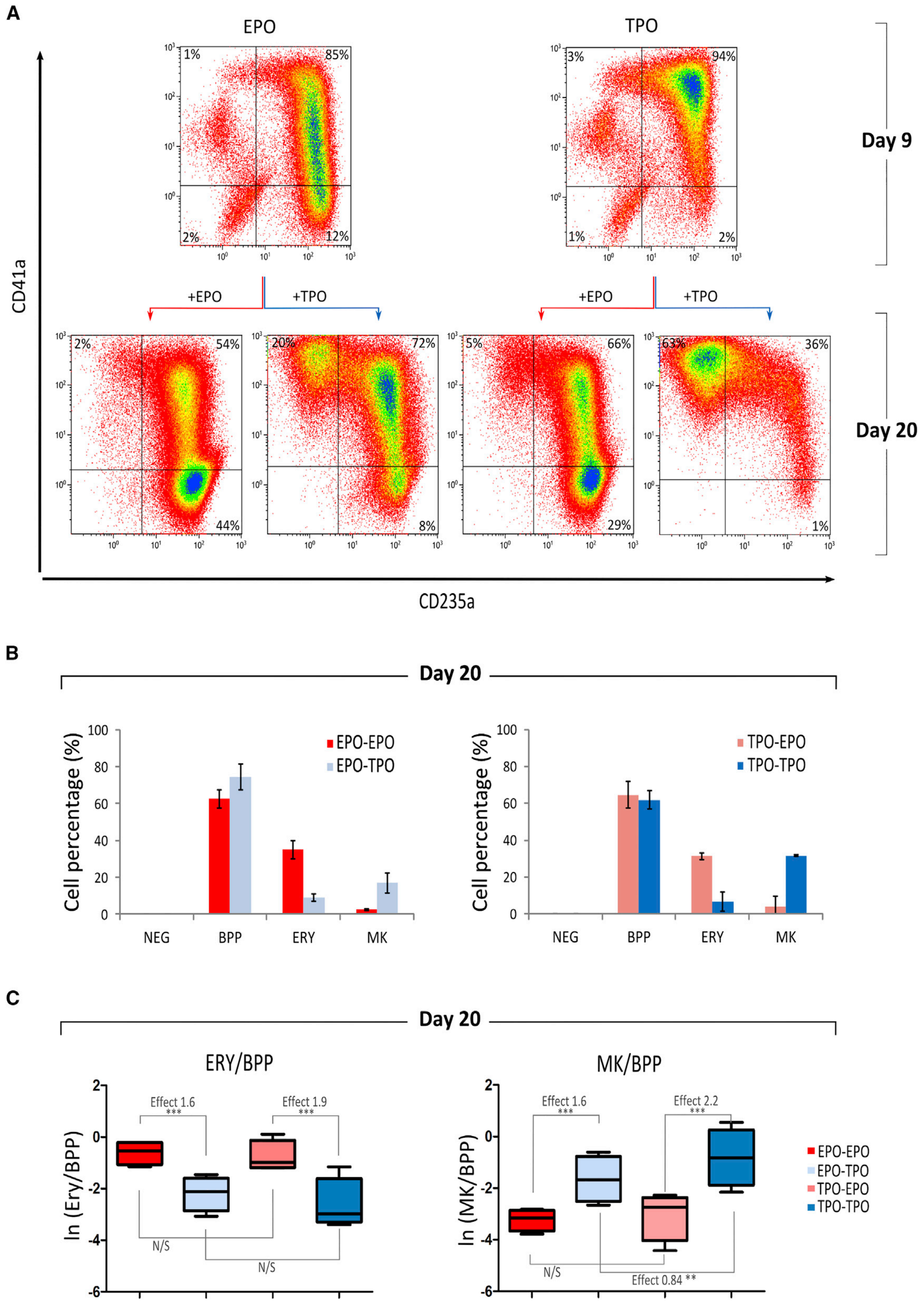

(legend on next page)

LSSmOrange for FLI1 (plasmid maps in Supplemental Experimental Procedures). Each reporter and TF pair is expressed from a single mRNA embedding an E2A self-cleaving peptide sequence. Using the flow cytometry settings and gating strategy shown in Figures 4A–4C, single-cell MKs were sorted according to each individual transgene signature: G(ATA1)+ or –, F(LI1)+ or – and T(AL1)+ or –. Single-cell RNA sequencing confirmed the correlation between protein fluorescent signal detected by flow cytometry and transgene transcript expression level (Figure 4D). The reporter fluorescence intensity measured by flow cytometry therefore gives a direct reading of the expression level of the corresponding TF transgene in each individual cell (Figure 5A) (Kim et al., 2011).

We monitored the evolution of the transgene expression patterns over the course of the culture. Remarkably, transgene patterns in the whole culture were highly similar in both EPO and TPO conditions up to day 9, with a clear diversification from that point onward (Figure 5B). This is in line with the minimum impact of the early cytokine treatment reported above, and supports the hypothesis of the early differentiation events being controlled primarily by transgene expression.

At day 2 post-transduction, 87% ± 4% of the cells co-expressed the three transgenes (GATA1, FLI1, and TAL1, from now on GFT) (Figure 5C, left panel). At day 9 of culture a proportion of cells had lost expression of one or several transgenes (Figure 5C). The cells that did not differentiate (no expression of CD41a nor CD235a) either expressed mostly TAL1 or did not show expression of any transgene (data not shown). The BPPs showed two predominant combinations of transgene expression: either the 3TFs were expressed (GFT) or only GATA1 and TAL1 (GT) (Figure 5C, middle panel). Crucially, this pattern was not statistically different between the BPPs grown in either EPO or TPO: 38% ± 5% versus 49% ± 5% of BPPs co-expressed GFT ($p > 0.25$), while 53% ± 5% versus 42% ± 4% showed the GT pattern in EPO and TPO, respectively ($p > 0.45$) (Figure 5C). Notably, the GT profile was dominant in the ERY population independently of the early cytokine treatment (75% ± 2% and 73% ± 1% of ERYs from EPO and TPO cultures, respectively; $p > 0.5$). Conversely, among the MK population, the main transgene expression pattern was GFT (46% ± 11% and 57% ± 15% in EPO and TPO, respectively; $p > 0.5$).

At day 20, irrespective of the cytokine conditions, the GFT pattern was predominant in the BPPs (56% versus 60% in EPO and TPO, respectively), while the ERYs were mostly lacking FLI1 transgenic expression (GT and T pattern dominating) and the MKs were enriched for the FLI1-containing transgenic patterns GFT or FT (Figure 5C, right panel).

These experiments were repeated with a further two iPSC lines and one ESC line which showed the same dynamics: expression of all three transgenes at day 2 and gradual emergence of two main cell subgroups: GFT cells in the TPO condition and GT cells in EPO (Figure 6A). In keeping with the faster dynamic of cell differentiation reported above (Figure 3B), the diversification of TF expression pattern between the two culture conditions also happened earlier than day 9. Crucially, regardless of the cytokines used in culture, GFT cells were again enriched within the MK population, while GT cells represented the majority of ERYs (Figure 6B).

These data showed that the transgenic TF pattern was the main determinant of the cell identity through FoP. The BPPs expressed mostly two transgenic patterns: GT and GFT. The MKs showed a bias for expression of all 3TFs (GFT), while the ERYs were biased toward expression of GT. From the results above we calculated that the likelihood of a differentiated non-BPP cell with high FLI1 expression to be an MK (positive predictive value) is >70% irrespective of the cytokine used. Similarly, the positive predictive value for the lack of FLI1 expression to identify ERY among differentiated non-BPP cells was >80% in both TPO and EPO.

Figure 3. The impact of Cytokines Used in the Early and Late Phases of Culture on Erythroid and MK Differentiation

Cells were FoP in EPO or TPO until day 9 (early phase) and then maintained in the same medium or switched to the other cytokine condition for the second phase (days 9–20). The impact on MK and ERY differentiation was monitored by flow cytometry at days 9 and 20.

(A) Representative flow cytometry dot plots for CD41a and CD235a expression on days 9 and 20 of FoP following culture regimens indicated by arrows.

(B) Percentages of cells at day 20 belonging to the NEG (CD41−/CD235−), BPP (CD41+/CD235+), ERY (CD41-/CD235+), and MK (CD41+/CD235−) populations, respectively (n = 4, mean ± SEM).

(C) Boxplots over sets of four replicates of the log(e) of the number of ERYs (left plot) and MKs (right plot) over the number of BPPs as detected by flow cytometry at day 20, broken down by cytokine exposure at days 9 and 20. The mean effect of TPO used between days 9 and 20 on a cell being an MK rather than BPP was similar (2.2 and 1.6) for cells cultured in TPO and EPO between days 2 and 9, respectively (***$p < 0.001$). The mean effect of EPO used between days 9 and 20 on a cell being an ERY rather than BPP was similar too (1.9 and 1.6) for each of the condition used between days 2 and 9, respectively (***$p < 0.001$). By contrast, conditional on the second cytokine, the effect of the cytokine used between days 2 and 9 on the MK/BPP and ERY/BPP were not significant in three of the four comparisons and below one for the fourth.

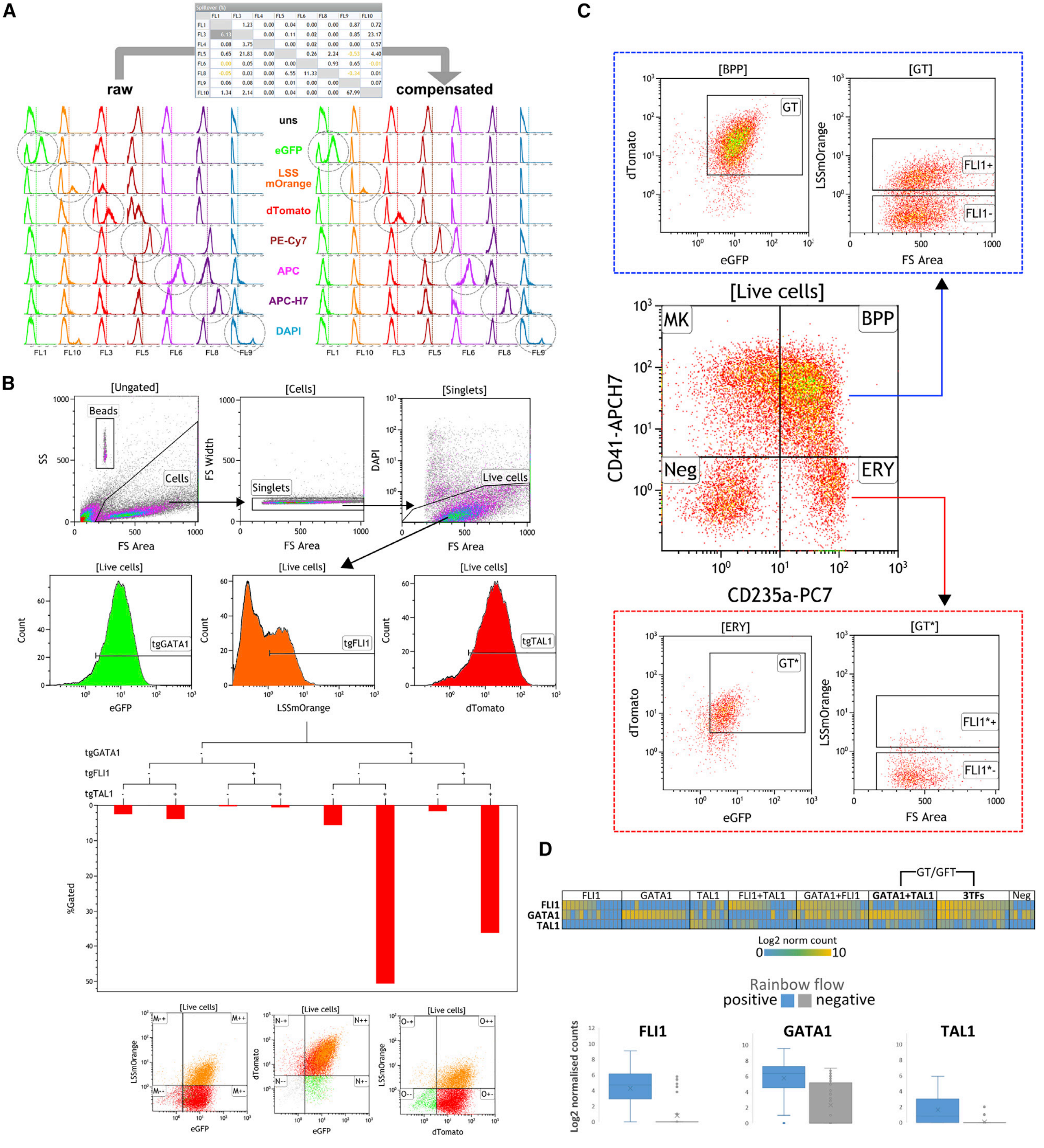

Figure 4. Rainbow Analysis by Flow Cytometry Faithfully Reports Levels of 3TF Transgenic Expression

Human iPSCs were transduced with the three rainbow vectors designed to report transgene expression and analyzed by multicolor flow cytometry for expression of eGFP(GATA1), LSSmOrange(FLI1), and dTomato(TAL1), in addition to the surface markers Pe-Cy7(CD235a), APC(CD309/CD42a), and APC-H7(CD41), and the DNA stain DAPI (viability). Representative data shown from the FFDK cell line at day 9 post-transduction.

(A) Single-color control samples were run alongside each flow experiment to set up a compensation matrix efficiently correcting for spillover between optical channels.

(legend continued on next page)

Expression of the FLI1 Transgene Dictates Lineage Commitment in BPPs

Based on these results, we hypothesized that FLI1 plays a pivotal role in the lineage commitment: GFT BPPs are destined to become MKs, while GT BPPs are destined to the ERY lineage regardless of the cytokines used in the culture medium. To demonstrate the instructive role of FLI1, day 9 BPPs from the EPO condition were sorted according to the transgene expression pattern (GFT versus GT, which together represent 90% of all BPPs, Figure 5C). Analysis by qRT-PCR confirmed that FLI1 total expression (transgene and endogene) was markedly lower in the GT versus GFT population (Figure 7A). The sorted GT and GFT BPPs were seeded into clonogenic assays. The distribution of colony types differed significantly between GT and GFT cells ($p < 0.001$). Again, MIXED colonies were rarely seen for each of these two populations, but GT cells produced a majority of ERY colonies (78% ± 10%), while GFT cells produced mainly MK colonies (62% ± 2%) (Figure 7B).

We reproduced these results in a further three hPSC lines transduced with the rainbow vectors. GT BPPs sorted at day 9 had barely any MK potential, while it was clearly present in the GFT BPPs (Figure S5).

Early Expression of FLI1 and Subsequent Silencing Is Necessary for ERY FoP

The lack of FLI1 expression in the erythroid-biased GT BPP cells at day 9 could either come from gene silencing in the cells that co-expressed all 3TFs early on in culture (>85% of the cells at day 2) or from the selective emergence of the few cells that were transduced with only GT from the start.

Lentiviral FLI1 genomic insertion was assessed in genomic DNA isolated from GT and GFT sorted BPPs at day 9. Remarkably, the relative number of FLI1 proviral integrations was similar in both populations, while the untransduced control cells showed no proviral integration (Figure 7C). This demonstrates that the GT BPPs actually emerged from a triple transduced GFT sub-population where specific silencing of the FLI1 transgene took place in the early stage of programming.

We previously showed that the presence of FLI1 (alongside GATA1 and TAL1) is necessary for the development of MKs from hPSCs in a TPO-containing medium (Moreau et al., 2016). However, the question remained whether this is also true for the programming of ERYs in EPO.

We therefore performed an experiment where cells were transduced with either all 3TFs or with GATA1 and TAL1 only (2TFs) and cultured in EPO. The number of viable cells was reduced after transduction with 2TFs at day 9 and significantly so at day 20 (Figure 7D, left panel). This was accompanied by a major reduction in the percentage of BPPs at day 9 from >62% ± 6% to 21% ± 4% (mean ± SEM) in 3TFs versus 2TFs, respectively (Figure 7D, right panel; $p < 0.01$). By day 20, there were barely any viable cells in the 2TFs condition but the majority of the 2TFs programmed cells were CD235a+ CD41a− ERYs. These few ERYs were, however, similar to those derived from the 3TFs culture in terms of morphology, hemoglobinization, and expression of surface markers such as Band 3 and RhD (data not shown).

DISCUSSION

We have previously demonstrated that the overexpression of 3TFs GATA1, FLI1, and TAL1 in hPSCs generates MKs at high purity (Moreau et al., 2016). In this manuscript, we show that FoP of hPSCs with these 3TFs actually leads to the formation of MK and ERY progenitors (BPPs) able to differentiate to ERYs or MKs when cultured in EPO or TPO condition, respectively. We show that lineage commitment of the BPPs happens early on during the cell differentiation. This is independent of the cytokines used in the first phase of culture, while the cytokines used in the second phase of culture facilitate the expansion and maturation of the already committed BPPs. We show that the initial expression of all 3TFs is essential to the FoP and we demonstrate that the sustained expression of the FLI1 transgene is the key molecular switch dictating MK versus ERY lineage bias.

(B) Gating strategy for analysis of the rainbow sub-populations. Single-color histograms were used to read each rainbow color separately from the single live cell gate. The statistics for the eight unique transgenic populations were then collected using the radar plot capacity of the Beckman Coulter Kaluza software.

(C) Flow cytometry sorting gating strategy for day10 cells. The [BPP] population was defined from the single live cell gate as cells co-expression the surface markers CD41 and CD235. The [BPP] population was further gated for cells co-expressing the reporters eGFP(GATA1) and dTomato(TAL1) further split into LSSmOrange(FLI1) positive and negative cells: this led to the collection of the [BPP-GFT] and [BPP-GT] populations, respectively, for the downstream analyses described in the manuscript. Illustration of the rainbow expression profile in the [ERY] population is also shown as a contrasting profile.

(D) The expression of the transgenic TFs from flow-sorted populations based on rainbow reporter expression was further analyzed at the transcript level and single-cell resolution by RNA sequencing. The Log2 normalized transgenic read counts from the eight possible rainbow combinations are shown as a heatmap (n = 111 cells). The boxplots show pooled data from rainbow-positive (blue) or -negative (gray) cells taking in consideration a single rainbow reporter at a time from the same dataset.

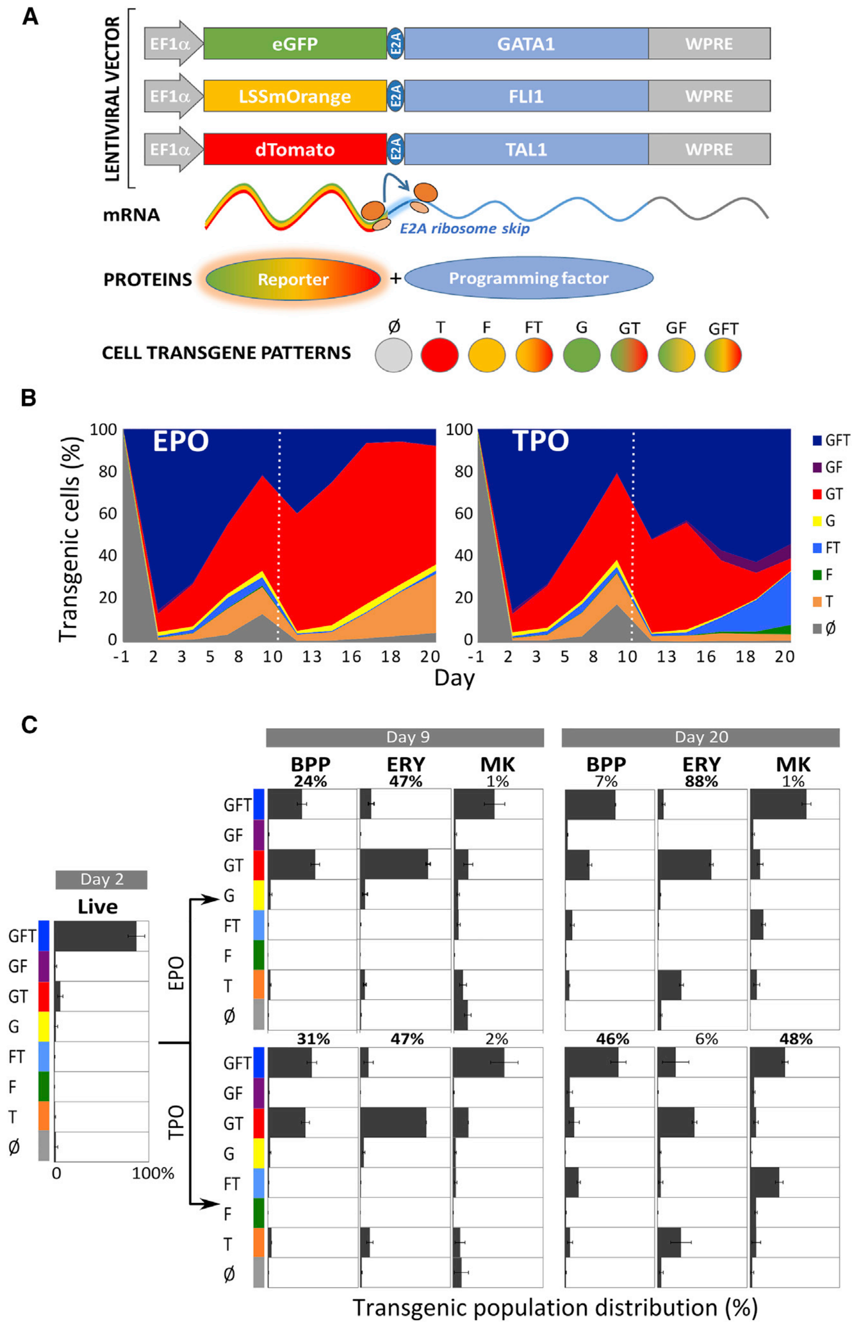

Figure 5. Bipotent Progenitors, Megakaryocytes, and Erythroblasts Have Consistent Transgene Expression Patterns Regardless of the Culture Cytokines

Forward programming was performed using rainbow vectors and transgene expression patterns were identified by flow cytometry.

(A) Schematic representation of the rainbow vector system. The open reading frame (GATA1, FLI1, and TAL1) for each transcription factor was cloned downstream of a different reporter gene (eGFP, LSSmOrange, and dTomato, respectively) separated by a self-cleaving E2A sequence (see vector maps in Supplemental Experimental Procedures). This ensures the production of the reporter, and programming transcription factor proteins are proportional since they are derived from a single mRNA. The fluorescence directly reports the programming factor expression in every single cell (see Figure S5).

(B) Representative depiction of the dynamic evolution of transgene expression patterns in viable cells from the hPSC stage (day −1) until day 20 post-transduction in EPO (left) and TPO (right) conditions. Cells were gated according to expression of G(ATA1), F(LI1), and T(AL1).

(C) FoP cell surface markers expression and transgene expression were analyzed by flow cytometry at day 2 (left column), day 9 (middle column), and day 20 (right column) post-transduction (EPO cultures in the top panel and TPO cultures in the bottom panel). Cells have been divided according to expression of differentiation markers (CD235a and CD41a) into BPP (CD41a+ CD235a+), ERY (CD235a+ CD41a−), and MK (CD235a− CD41a+). Transgene expression patterns (G and/or F and/or T) are shown as mean% ± SEM (n = 3).

A substantial body of work has been published showing that the ERY and MK lineages are closely related and that both emerge from a common megakaryocyte erythroid progenitor (MEP), which itself descends from a common myeloid progenitor (CMP) (Debili et al., 1996). CMPs also produce cells from the myeloid lineage (monocytes and polymorphonuclear cells) (Miyawaki et al., 2017). We have previously shown that, during FoP with FLI1, GATA1, and TAL1, hPSCs will differentiate into cells that express mesoderm and then hemogenic endothelium markers before differentiating into MKs (Moreau et al., 2016). This suggested that, rather than enforcing a direct jump to MK identity from the hPSC, FoP guides differentiation along the "normal" developmental pathway. However the 3TFs driving the FoP impose a restriction on lineage options so that FoP cells never form the full array of myeloid cells. We show here that FoP with 3TFs generates a population of cells capable of forming both ERYs and

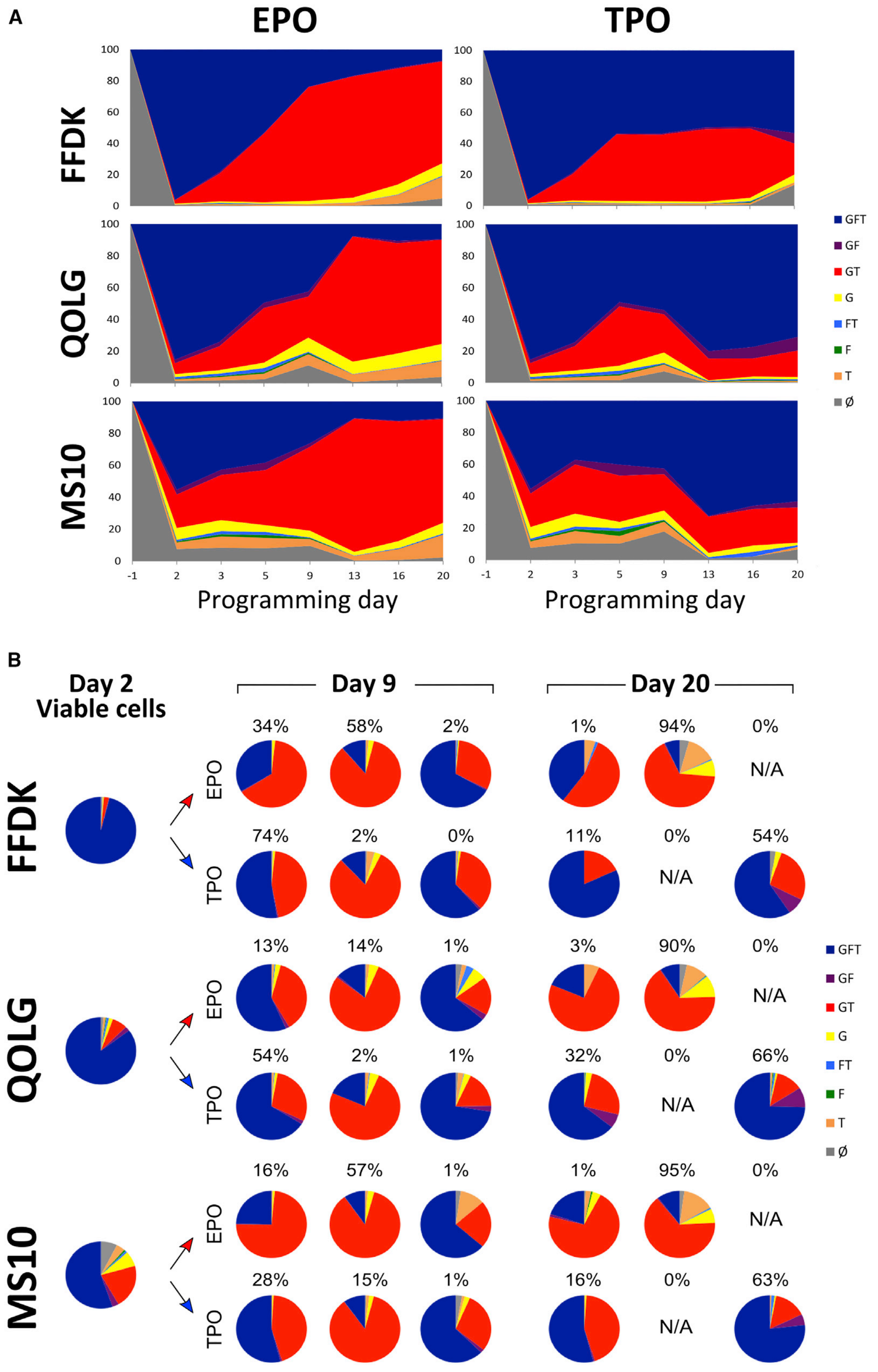

(legend on next page)

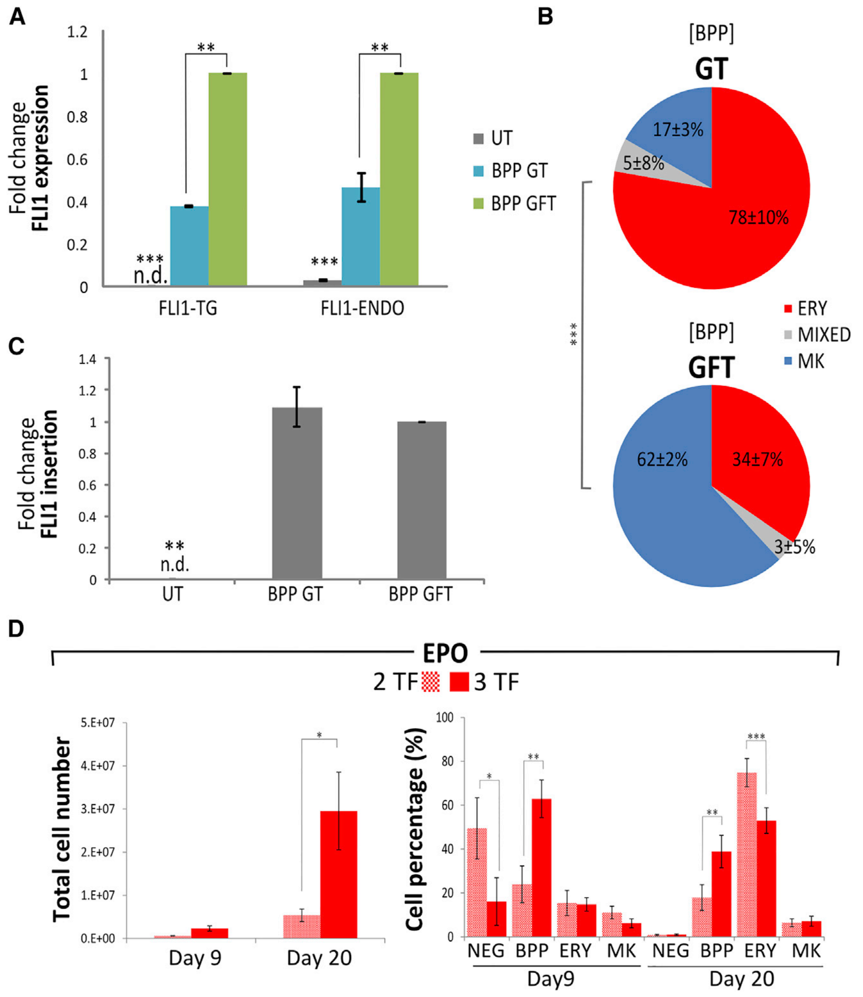

Figure 7. Changes in FLI1 Transgene Expression Dictates Forward Programming Outcome

Day 9 CD41+ CD235a+ BPPs were sorted by flow cytometry into GATA1+/FLI1+/TAL1+ (GFT) and GATA1+/FLI1−/TAL1+ (GT) populations.

(A) FLI1 transgene and endogenous expression was measured by qRT-PCR on sorted populations and normalized to the GFT population; GFT (green bars), GT (blue bars), and untransduced (gray bars).

(B) The clonogenic potential of the GFT and GT-sorted BPPs cells was tested by CFU assay. The pie charts show colony distribution from both populations after 14 days (n = 2, mean%); erythroid colonies (ERY, red), megakaryocyte-erythrocyte mixed colonies (MIXED, gray), and megakaryocyte colonies (blue). Poisson regression analysis shows that the distribution of the number of colonies per cell type depends strongly on the set of expressed TFs (***$p < 2.2 \times 10^{-16}$).

(C) Detection of integrated FLI1 provirus by qPCR in the genomic DNA of day 9 GFT and GT-sorted BPPs; UT, untransduced cells (n = 2, mean% normalized to GFT cells).

(D) Forward programming was performed using 3TFs (GATA1, FLI1, and TAL1) or 2TFs (GATA1 and TAL1) in EPO culture conditions. Cells were monitored by flow cytometry at days 9 and 20 post-transduction to determine viable cell numbers (left) and the CD41a/CD235a cell phenotype (right) (n = 5, mean% ± SEM). Cell numbers were significantly decreased with 2TFs as well the overall proportion of BPPs generated at days 9 and 20.

MKs, in keeping with these cell types' close developmental relationship.

Evidence has recently emerged over that has thrown a somewhat different light upon the "established" hierarchical hematopoietic model. Self-renewing MK-biased progenitor cells have been identified as high up the hierarchical tree as HSCs (Notta et al., 2016; Sanjuan-Pla et al., 2013), and the existence of unipotent MK-restricted progenitors has been demonstrated among the CMP population. With the emergence of single-cell biology, it has become clearer that even highly purified populations of HSCs or progenitors, which appear phenotypically homogeneous based on cell surface markers, are actually often molecularly and functionally heterogeneous, with a different lineage assignment within each individual cell. This model also seems to apply to MEPs isolated from primary hematopoietic tissue. Multiple studies of these MEPs have shown that, although at bulk level these cells have a phenotype

Figure 6. Pattern of Transgene Expressions during Differentiation in EPO and TPO

Three additional cell lines are presented here, additional to the one presented in Figure 4. FFDK and QOLG are iPSC lines, MS10 is an ESC line. The cells were forward programmed with the rainbow vectors and split at day 2 into EPO or TPO cultures.

(A) Representative depiction of the dynamic evolution of transgene expression patterns in viable cells from the hPSC stage (day −1) to day 20 post-transduction in EPO (left) and TPO (right) conditions. Cells were gated according to expression of G(ATA1), F(LI1), and T(AL1).

(B) FoP cell surface marker expression and transgene expression were analyzed by flow cytometry at day 2 (left panel), day 9 (middle panel), and day 20 (right panel) post-transduction (EPO cultures in the top line and TPO cultures in the bottom line). Cells have been divided according to expression of differentiation markers (CD235a and CD41a) into BPP (CD41a+ CD235a+), ERY (CD235a+ CD41a−), and MK (CD235a− CD41a+). Transgene expression patterns (G and/or F and/or T) are shown in the pie charts.

suggesting bipotentiality, they are rarely capable of forming truly mixed colonies, where both ERYs and MKs are produced from a single cell (Debili et al., 1996; Papayannopoulou et al., 1996; Pronk et al., 2007). Instead, the vast majority of single-cell MEP seeding experiments have shown restricted differentiation biased toward either ERYs or MKs. It is possible that the lineage fate decision among cells that appear phenotypically identical is based on variations in TF networks. Our data provide evidence supporting this. The BPP cells harvested at day 9 post-transduction show properties similar to MEPs isolated from primary bone marrow tissue: although these the phenotype of these cells suggests bipotentiality (they express both CD235a and CD41a), the clonogenic assays showed that the vast majority of these cells are already committed to either one or the other differentiation pathway. Crucially, this fate decision is made very early on during the programming, as this unilineage bias was demonstrated in the CD235a+ CD41a+ cells as soon as they emerged during differentiation at day 5 post-transduction. True functional bipotentiality is therefore likely to be a very transient stage.

To unravel the molecular basis for the dissociation between the expression of cell surface markers (which suggests bipotentiality) and individual cell fate decision (which suggests early unilineage restriction), we used rainbow vectors in combination with clonogenic assays. This allowed us to demonstrate that cell fate decision is made on the basis of the expression of the FLI1 transgene. It has long been known that FLI1 (together with KLF1) acts as a toggle switch to dictate whether a cell goes toward the MK or ERY pathway (Starck et al., 2003; Psaila et al., 2016). The existence of this toggle switch in forward-programmed cells is reinforced by the fact that, when we measured KLF1 expression in the sorted BPP cells, we found that KLF1 was overexpressed 3-fold in GT versus GFT cells (data not shown).

Intriguingly, >85% of the cells expressed all 3TFs in the period immediately following transduction and the BPP cells lacking FLI1 expression later in the culture were confirmed to have been transduced by the FLI1 viral vector. Following the concurrent expression of all 3TFs in most cells at day 2, unique silencing events in each individual cell leads to the formation of a population of cells with a mosaic pattern of TF expression. FLI1 expression dictated subsequent differentiation potential. Although ERYs were produced after FoP with TAL1 and GATA1 only, and although those were phenotypically similar to those produced after FoP with the 3TFs, the cell number output dropped by an order of magnitude. We have previously shown that hPSCs forward programmed with the 3TFs toward the MK lineage go through a hemogenic endothelium stage. Using FLK1 as a marker of hemogenic endothelium, we show that ERY differentiation also goes through the same stage of differentiation, with peak of expression seen at days 2–3 post-transduction in both TPO and EPO cultures (Figure S6A). However, using the rainbow vectors we show that a much smaller proportion of GT cells expressed FLK1 compared with GFT cells (Figure S6B). Given the small number of ERYs obtained with 2TFs FoP, we hypothesize that only cells that go through a hemogenic endothelium phase are ultimately able to differentiate into mature ERYs, and for this to happen early expression of all 3TFs is required.

We conclude that the transient expression of FLI1 is necessary to confer proliferative ability to the FoP cells, possibly through the activation of the hemogenic endothelium program, but its subsequent silencing is necessary in order for the FoP cell to commit to the erythroid lineage. How long the FLI1 transgene needs to be expressed in order to have this effect, and how this effect is mediated at the molecular level, remains an open question. Inducible systems have now been used in order to overexpress TFs to produce somatic cells from hPSCs without the need for lentiviral vectors (Pawlowski et al., 2017). However, our findings illustrate that the change of expression of the different TFs during differentiation may have a marked influence on the somatic cell output, and this is something to bear in mind if, for example, an inducible system was used to drive FoP rather than a lentiviral-based approach. Recent papers have also suggested that the maintenance of expression of FLI1 may have a crucial effect on the quality of MKs generated by cytokine-driven differentiation of primary HPCs or hiPSCs, particularly their ability to form platelets (Hirata et al., 2013; Vo et al., 2017).

Recent publications have demonstrated the production of bona fide transplantable HSCs from hPSCs and mouse adult endothelial cells (Lis et al., 2017; Sugimura et al., 2017). In both cases, cells were FoP with a cocktail of TFs, but crucially needed niche signals in order to mature into HSCs (either post-injection into a murine recipient or by co-culturing on endothelial cells). This raises the question of "nurture" versus "nature." The output of HSCs in these experiments was low, suggesting that perhaps FoP by itself did not enforce cell identity, but made cells responsive to their environment in order to become an HSC. By contrast, in our own FoP experiments, we show that the priming toward cell identity in the early stages of the culture was very efficient, and that lineage commitment was independent of the cytokines the cells were grown in, but, instead, was primarily driven by the expression of the FoP TFs. Once the BPP emerged and cells were committed to either lineage, the cytokines provided the signal allowing these cells to expand and mature according to their already established fate decision. It is possible that the contrast between our results with erythroid/MK progenitors and those with hPSC-derived HSCs is due to the fact that to create

HSCs the TFs used intervene "early" in the developmental pathway, where cells may need nurturing by their environment. In contrast, we use TFs that enforce cell identity further along the developmental pathway toward a terminally differentiated cell type where the nurturing environment may have a less prominent role.

The FoP hPSC-derived ERYs were shown to be capable of maturing to the normoblast stage, but enucleated reticulocytes were rarely observed. Similar results have been obtained in other hPSC-based red cell culture systems (including those relying on directed differentiation), and this has been attributed to the "embryonic" identity of hPSC-derived ERYs (Olivier et al., 2016; Yang et al., 2017). In keeping with this hypothesis, the FoP ERYs mostly expressed the fetal globin chain (γ) together with embryonic globins (ε). Manipulation of the chemical environment of hPSCs at early stages of differentiation (namely Wnt and activin signaling) has been shown to switch hematopoietic cell differentiation from the embryonic to the definitive pathway (Sturgeon et al., 2014). It may be that combining both this chemical approach with the high efficiency of our FoP process would allow the production of large quantities of pure ERYs expressing adult globins capable of enucleating efficiently. The use of chemically defined media, reduced cytokine cocktails and cell manipulation, coupled with the purity and high number of ERYs obtained, make our FoP process stand out from other protocols seeking to derive ERYs from hPSCs. The FoP approach may one day produce clinically relevant commercially viable quantities of red cells for transfusion from hPSCs.

EXPERIMENTAL PROCEDURES

Forward Programming

Sub-confluent hiPSC cultures were dissociated to small clumps using 0.5 mM EDTA DPBS. On transduction day, lentiviral particles were added to mesoderm medium CDM containing protamine sulfate (Moreau et al., 2016). After 24 hr, cells were washed and fresh mesoderm medium added without protamine sulfate. Twenty-four hours later, culture medium was changed to either ERY or MK medium, with half-volume renewal every 3 days. At day 9 post-transduction, floating cells were collected from the supernatant and pooled with the adherent fraction of the culture dissociated to single cells using TrypLE (Thermo Fisher Scientific). The single-cell preparation was further cultivated in suspension at 2×10^5 cells/mL for an additional 11 days in ERY or MK medium, including half medium renewal every 3 days and 1:4 cell split when cultures reached $>1 \times 10^6$ cells/mL.

Cord Blood ERY Culture

CD34+ haematopoietic progenitors were isolated from human CB samples obtained as part of an ethically approved study with informed consent, and cells were differentiated as described previously (Griffiths et al., 2012).

Flow Cytometry Analysis

Single-cell suspensions were stained for 20 min at room temperature using combinations of FITC-, PE-, PE-Cy7-, APC-, and APC-H7-conjugated antibodies (Table S1). Background fluorescences were set against fluorochrome-matched isotype control antibodies and compensation matrices defined using single color-stained cells (Figure 4). Enucleation was assessed by DRAQ5 (BioLegend) stain following manufacturer's instructions.

Colony-Forming Unit Assays

The clonogenic potential of FoP cultures was assessed in semi-solid methylcellulose medium supplemented with serum, EPO, stem cell factor, interleukin-3 (IL-3), IL-6, granulocyte colony-stimulating factor, and granulocyte-macrophage colony-stimulating factor (STEMCELL Technologies, H4435). The quantitative and qualitative colony outcome was monitored after 14 days upon blind microscopic observation from at least two people and results averaged.

Gene Expression Analysis by qRT-PCR

Total RNA was extracted using RNeasy kits (QIAGEN) according to the manufacturer's instructions, including DNase treatment. qRT-PCR primer pairs (Table S2) were designed to amplify cDNA only, detect all known isoforms, and have no reported off-target matches against the human NCBI RefSeq database. All primers were tested before use for 80%–120% PCR efficiencies and single dissociation curves. We used UTR priming (absent from transgene sequences) to specifically monitor endogene expression, while transgene expression was selectively measured using a common reverse primer specific to the viral vector sequence.

Cell Morphology

Single cells ($1–2 \times 10^5$) in suspension were spun onto a glass slide using cytofunnels and stained using the Giemsa (VWR) staining method.

Western Blotting

Cells were lysed in radioimmunoprecipitation assay buffer containing proteinase inhibitors (complete, Roche) and sonicated for 5 min. Samples were then resolved by SDS-PAGE and transferred to a polyvinylidene difluoride membrane (Millipore), which was blocked with 10% milk and incubated for 1 hr in specific antibodies for α-, β-, γ-, and ξ-globin and β-actin characterization (Table S1).

Statistics

Regression Analysis

The data for the colony-forming unit experiments were analyzed using mixed effects Poisson regression while flow cytometry data were analyzed using mixed effects logistic regression.

General Analysis

Results are presented as mean ± SEM or SD, with n representing the number of biological replicates. The p values derived from applying either two-tailed Student's t tests of a difference in means between two samples or one-way ANOVA Tukey *post-hoc* test are given.

Statistical significances are indicated in the figures as follows: *p < 0.05, **p < 0.01, ***p < 0.001.

Further details included in the Supplemental Information document.

SUPPLEMENTAL INFORMATION

Supplemental Information includes Supplemental Experimental Procedures, six figures, and two tables and can be found with this article online at https://doi.org/10.1016/j.stemcr.2018.11.001.

AUTHOR CONTRIBUTIONS

A.D. and J.B.-B. designed and performed the experiments and wrote the manuscript. C.L., A.M., N.F., and A.E. designed and performed the experiments. E.T. performed statistical regression analyses. J.B. performed the bioinformatics analyses for the single-cell sequencing. T.M., M.R.T., and C.G. supervised the work, designed experiments, and wrote the manuscript.

ACKNOWLEDGMENTS

We acknowledge funding from the BHF Cambridge Centre of Excellence (RE/13/6/30180), the Wellcome Trust (Novosang consortium), and the NHS Blood and Transplant Service. We are grateful for the intellectual support and practical help from Dr Jan Frayne (University of Bristol), Dr Jo Mountford (Scottish National Blood Transfusion Service), Dr Ashley Toye (University of Bristol), Dr Lila Diamanti, and Prof Bertie Gottgens (University of Cambridge Stem Cell Institute). We acknowledge SMART's graphical art (Servier Medical Art by Servier, https://smart.servier.com), licensed under a Creative Commons Attribution 3.0 Unported License and used in the graphical abstract.

Received: March 23, 2018
Revised: November 1, 2018
Accepted: November 1, 2018
Published: November 29, 2018

REFERENCES

Ali, A., Auvinen, M.K., and Rautonen, J. (2010). The aging population poses a global challenge for blood services. Transfusion *50*, 584–588.

Debili, N., Coulombel, L., Croisille, L., Katz, A., Guichard, J., Breton-Gorius, J., and Vainchenker, W. (1996). Characterization of a bipotent erythro-megakaryocytic progenitor in human bone marrow. Blood *88*, 1284–1296.

Dore, L.C., and Crispino, J.D. (2011). Transcription factor networks in erythroid cell and megakaryocyte development. Blood *118*, 231–239.

Griffiths, R.E., Kupzig, S., Cogan, N., Mankelow, T.J., Betin, V.M., Trakarnsanga, K., Massey, E.J., Lane, J.D., Parsons, S.F., and Anstee, D.J. (2012). Maturing reticulocytes internalize plasma membrane in glycophorin A-containing vesicles that fuse with autophagosomes before exocytosis. Blood *119*, 6296–6306.

Han, G.C., Vinayachandran, V., Bataille, A.R., Park, B., Chan-Salis, K.Y., Keller, C.A., Long, M., Mahony, S., Hardison, R.C., and Pugh, B.F. (2016). Genome-wide organization of GATA1 and TAL1 determined at high resolution. Mol. Cell. Biol. *36*, 157–172.

Hirata, S., Takayama, N., Jono-Ohnishi, R., Endo, H., Nakamura, S., Dohda, T., Nishi, M., Hamazaki, Y., Ishii, E., Kaneko, S., et al. (2013). Congenital amegakaryocytic thrombocytopenia iPS cells exhibit defective MPL-mediated signaling. J. Clin. Invest. *123*, 3802–3814.

Hirose, S., Takayama, N., Nakamura, S., Nagasawa, K., Ochi, K., Hirata, S., Yamazaki, S., Yamaguchi, T., Otsu, M., Sano, S., et al. (2013). Immortalization of erythroblasts by c-MYC and BCL-XL enables large-scale erythrocyte production from human pluripotent stem cells. Stem Cell Reports *1*, 499–508.

Irion, S., Nostro, M.C., Kattman, S.J., and Keller, G.M. (2008). Directed differentiation of pluripotent stem cells: from developmental biology to therapeutic applications. Cold Spring Harb. Symp. Quant. Biol. *73*, 101–110.

Kim, J.H., Lee, S.R., Li, L.H., Park, H.J., Park, J.H., Lee, K.Y., Kim, M.K., Shin, B.A., and Choi, S.Y. (2011). High cleavage efficiency of a 2A peptide derived from porcine teschovirus-1 in human cell lines, zebrafish and mice. PLoS One *6*, e18556.

Kurita, R., Suda, N., Sudo, K., Miharada, K., Hiroyama, T., Miyoshi, H., Tani, K., and Nakamura, Y. (2013). Establishment of immortalized human erythroid progenitor cell lines able to produce enucleated red blood cells. PLoS One *8*, e59890.

Leach, L.L., and Clegg, D.O. (2015). Concise review: making stem cells retinal: methods for deriving retinal pigment epithelium and implications for patients with ocular disease. Stem Cells *33*, 2363–2373.

Lilly, M.A., Davis, M.F., Fabie, J.E., Terhune, E.B., and Gallicano, G.I. (2016). Current stem cell based therapies in diabetes. Am. J. Stem Cells *5*, 87–98.

Lis, R., Karrasch, C.C., Poulos, M.G., Kunar, B., Redmond, D., Duran, J.G.B., Badwe, C.R., Schachterle, W., Ginsberg, M., Xiang, J., et al. (2017). Conversion of adult endothelium to immunocompetent haematopoietic stem cells. Nature *545*, 439–445.

Miyawaki, K., Iwasaki, H., Jiromaru, T., Kusumoto, H., Yurino, A., Sugio, T., Uehara, Y., Odawara, J., Daitoku, S., Kunisaki, Y., et al. (2017). Identification of unipotent megakaryocyte progenitors in human hematopoiesis. Blood *129*, 3332–3343.

Moreau, T., Evans, A.L., Vasquez, L., Tijssen, M.R., Yan, Y., Trotter, M.W., Howard, D., Colzani, M., Arumugam, M., Wu, W.H., et al. (2016). Large-scale production of megakaryocytes from human pluripotent stem cells by chemically defined forward programming. Nat. Commun. *7*, 11208.

Notta, F., Zandi, S., Takayama, N., Dobson, S., Gan, O.I., Wilson, G., Kaufmann, K.B., Mcleod, J., Laurenti, E., Dunant, C.F., et al. (2016). Distinct routes of lineage development reshape the human blood hierarchy across ontogeny. Science *351*, aab2116.

Olivier, E.N., Marenah, L., Mccahill, A., Condie, A., Cowan, S., and Mountford, J.C. (2016). High-efficiency serum-free feeder-free erythroid differentiation of human pluripotent stem cells using small molecules. Stem Cells Transl. Med. *5*, 1394–1405.

Palmer, A.F., and Intaglietta, M. (2014). Blood substitutes. Annu. Rev. Biomed. Eng. *16*, 77–101.

Papayannopoulou, T., Brice, M., Farrer, D., and Kaushansky, K. (1996). Insights into the cellular mechanisms of erythropoietin-thrombopoietin synergy. Exp. Hematol. *24*, 660–669.

Pawlowski, M., Ortmann, D., Bertero, A., Tavares, J.M., Pedersen, R.A., Vallier, L., and Kotter, M.R.N. (2017). Inducible and deterministic forward programming of human pluripotent stem cells into neurons, skeletal myocytes, and oligodendrocytes. Stem Cell Reports *8*, 803–812.

Pronk, C.J., Rossi, D.J., Mansson, R., Attema, J.L., Norddahl, G.L., Chan, C.K., Sigvardsson, M., Weissman, I.L., and Bryder, D. (2007). Elucidation of the phenotypic, functional, and molecular topography of a myeloerythroid progenitor cell hierarchy. Cell Stem Cell *1*, 428–442.

Psaila, B., Barkas, N., Iskander, D., Roy, A., Anderson, S., Ashley, N., Caputo, V., Lichtenberg, J., Loaiza, S., Bodine, D., et al. (2016). Single-cell profiling of human megakaryocyte-erythroid progenitors identifies distinct megakaryocyte and erythroid differentiation pathways. Genome Biol. *17*, 83.

Sanjuan-Pla, A., Macaulay, I.C., Jensen, C.T., Woll, P.S., Luis, T.C., Mead, A., Moore, S., Carella, C., Matsuoka, S., Bouriez Jones, T., et al. (2013). Platelet-biased stem cells reside at the apex of the haematopoietic stem-cell hierarchy. Nature *502*, 232–236.

Starck, J., Cohet, N., Gonnet, C., Sarrazin, S., Doubeikovskaia, Z., Doubeikovski, A., Verger, A., Duterque-Coquillaud, M., and Morle, F. (2003). Functional cross-antagonism between transcription factors FLI-1 and EKLF. Mol. Cell. Biol. *23*, 1390–1402.

Stoker, T.B., and Barker, R.A. (2016). Cell therapies for Parkinson's disease: how far have we come? Regen. Med. *11*, 777–786.

Sturgeon, C.M., Ditadi, A., Awong, G., Kennedy, M., and Keller, G. (2014). Wnt signaling controls the specification of definitive and primitive hematopoiesis from human pluripotent stem cells. Nat. Biotechnol. *32*, 554–561.

Sugimura, R., Jha, D.K., Han, A., Soria-Valles, C., Da Rocha, E.L., Lu, Y.F., Goettel, J.A., Serrao, E., Rowe, R.G., et al. (2017). Haematopoietic stem and progenitor cells from human pluripotent stem cells. Nature *545*, 432–438.

Vo, K., Jarocha, D., Lyde, R., Hayes, V., Thom, C., Sullivan, S., French, D., and Poncz, M. (2017). FLI1 level during megakaryopoiesis affects thrombopoiesis and platelet biology. Blood *129*, 3486–3494.

Yang, C.T., Ma, R., Axton, R.A., Jackson, M., Taylor, A.H., Fidanza, A., Marenah, L., Frayne, J., Mountford, J.C., and Forrester, L.M. (2017). Activation of KLF1 enhances the differentiation and maturation of red blood cells from human pluripotent stem cells. Stem Cells *35*, 886–897.

Stem Cell Reports, Volume *11*

Supplemental Information

Transcription Factor Levels after Forward Programming of Human Pluripotent Stem Cells with GATA1, FLI1, and TAL1 Determine Megakaryocyte versus Erythroid Cell Fate Decision

Amanda Dalby, Jose Ballester-Beltrán, Chiara Lincetto, Annett Mueller, Nicola Foad, Amanda Evans, James Baye, Ernest Turro, Thomas Moreau, Marloes R. Tijssen, and Cedric Ghevaert

__Supplementary Figures__

Supplementary Figure 1: Mixed effect logistic regression analysis of the expression of CD41a and CD235a of forward programmed cells

A. Day 9 post-transduction

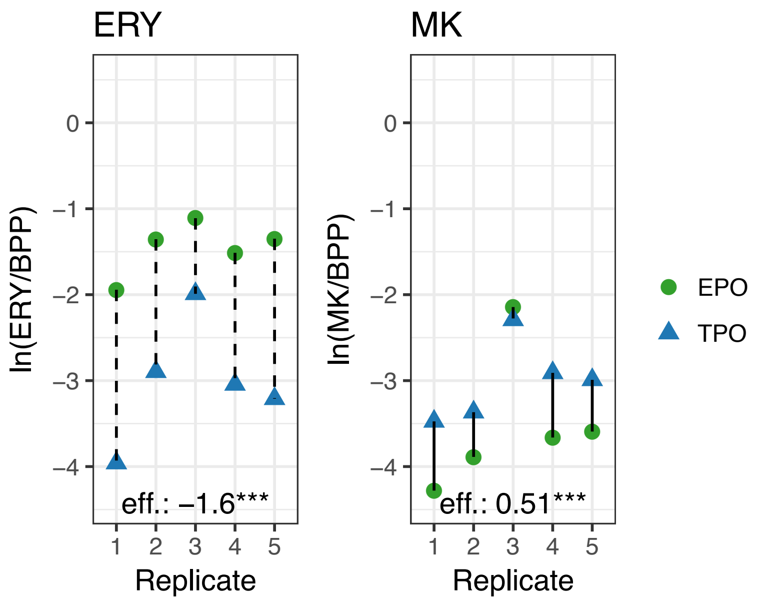

B. Day 20 post-transduction

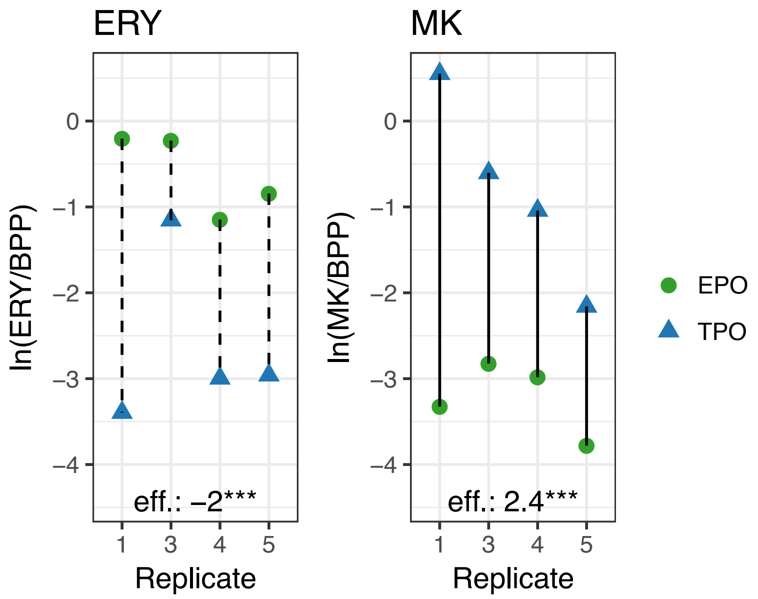

C. Cytokine Switch Experiment

First treatment held constant | **Second treatment held constant**

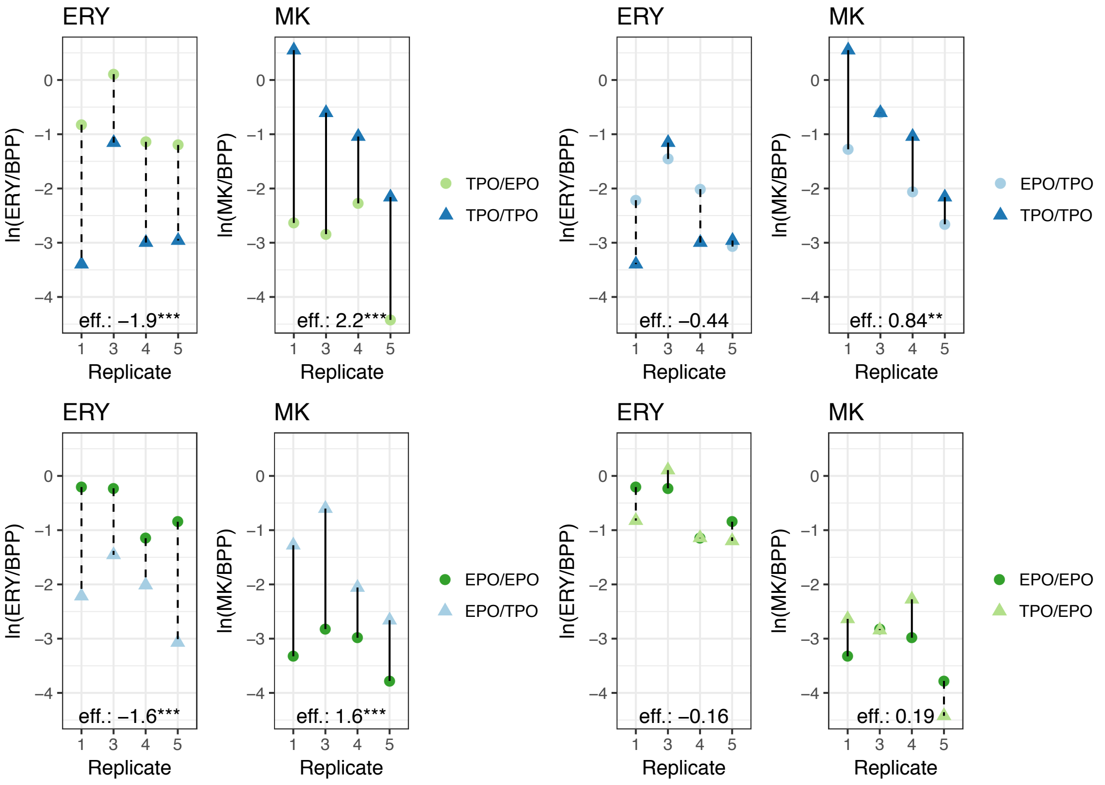

Supplementary Figure 1. Mixed effect logistic regression analysis of the expression of CD41a and CD235a of forward programmed cells

The flow cytometry data were analysed using mixed effects logistic regression. The binary outcome indicated whether a cell was CD41+CD235- (MK) or CD41+CD235+ (BPP) in one analysis and CD41-CD235+ (ERY) or CD41+CD235+ (BPP) in another analysis. This was performed for each replicate experiment of the cells harvested at day 9 (**A**), day 20 (**B**) where the same cytokine was used from day 2 till day 20 and for the cytokine switch experiment (**C**) where cytokine were switched at day 9 and data analysed at day 20. For each replicate the log(e) of the numbers of ERY or MK over the number of BPP is represented and color-coded according to the cytokine the cells were grown in. The cytokine "effect" is represented by the vertical line between the two cytokine conditions in each replicate. The models included a fixed effect for each replicate and random slopes with common variance representing the replicate-specific changes in the log odds due to cytokine exposure (e.g. TPO vs EPO). The random slopes accounted for evident variation in the treatment effects across replicates.

Supplementary Figure 2. CFU-assay colony characterization for myeloid lineages.

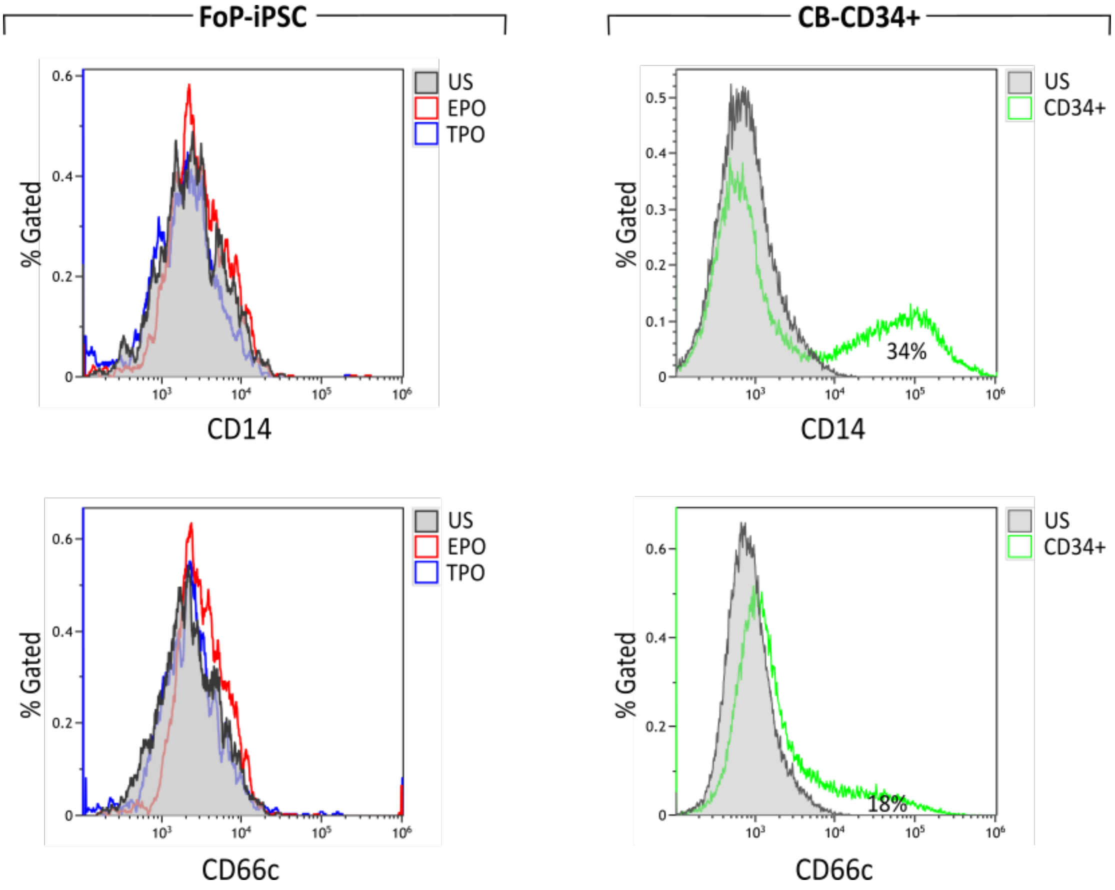

Supplementary Figure 2. CFU-assay colony characterization for myeloid lineages. The whole colony content of CFU assays was analysed by flow cytometry for CD14 (monocyte) and CD66c (granulocyte) expression, from day 9 forward programmed cells grown in EPO (red line) and TPO (blue line), or from CD34+ primary cord blood progenitors (green line); unstained cells (grey line).

Supplementary figure 3. Forward programming of additional hPSC lines to produce erythroblasts.

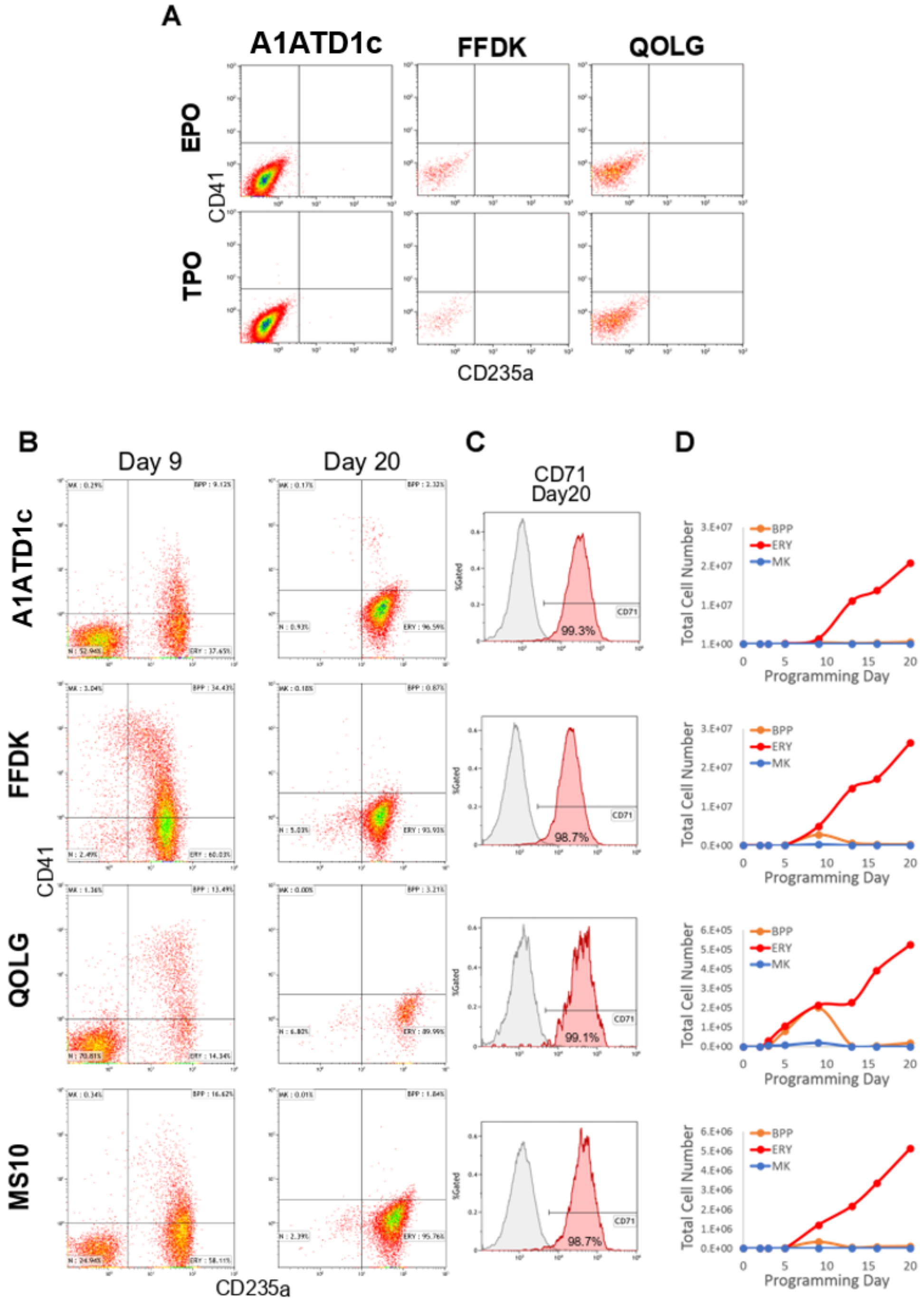

Supplementary figure 3. Forward programming of additional hPSC lines to produce erythroblasts. In addition to the iPSC line presented in the main figure (A1ATD1c), an additional 2 iPSC lines (FFDK and QOLG) and an ESC line were forward programmed with the 3TFs GATA1, TAL1 and FLI1 in EPO culture conditions. **(A)** Representative dot plots of untransduced cells for the expression of CD41a and CD235a after 9 days in EPO culture showing no evidence of cell differentiation towards the erythroid or MK lineage. **(B)** Representative flow cytometry dot plots for CD41a and CD235a expression on day 9 (left) and day 20 of culture (right) showing the presence of a population of cells expressing both markers at day 9 as well as single positive CD235a ERYs whilst the day 20 cells represent a pure ERY population. **(C)** Flow cytometry histogram showing CD71

expression of the forward programmed cells at day 20. **(D)** Graphs demonstrating the cell numbers accumulating over time according to cell type: BPP (CD235a+ CD41a+), ERY (CD235a+ CD41a-) and MKs (CD235a- CD41a+) starting from $3x10^4$ stem cells.

Supplementary Figure 4. Clonogenic assay with forward programmed cells seeded at day 5 post-transduction.

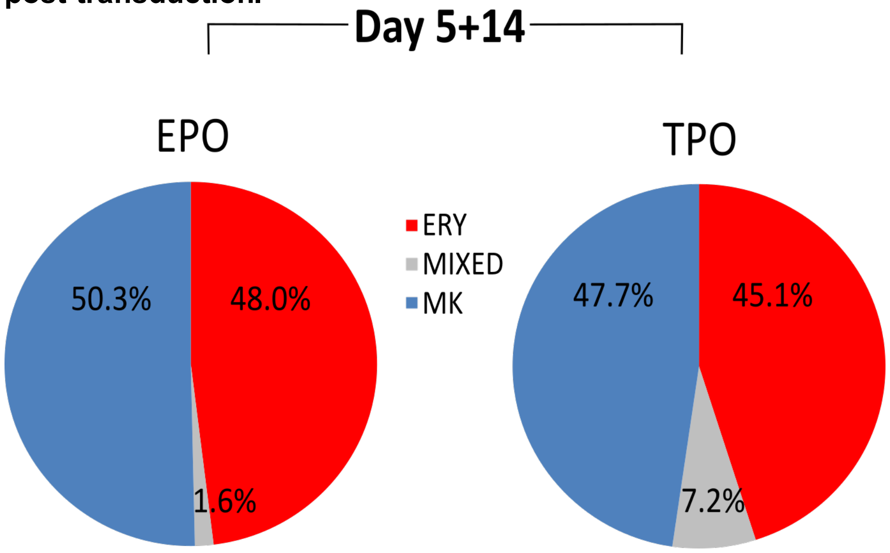

Supplementary Figure 4. Clonogenic assay with forward programmed cells seeded at day 5 post-transduction. Day 5 forward programmed cells cultured in either EPO (left) or TPO (right) were seeded into clonogenic assays and the colony types recorded after 14 days. Mixed colonies are rare (less than 10%) in both conditions and the number of MK or ERY colonies are equally balanced regardless of the cytokine the cells were grown in prior to seeding.

Supplementary Figure 5. Colony forming potential of day 9 BPPs sorted for GT or GFT transgene expression.

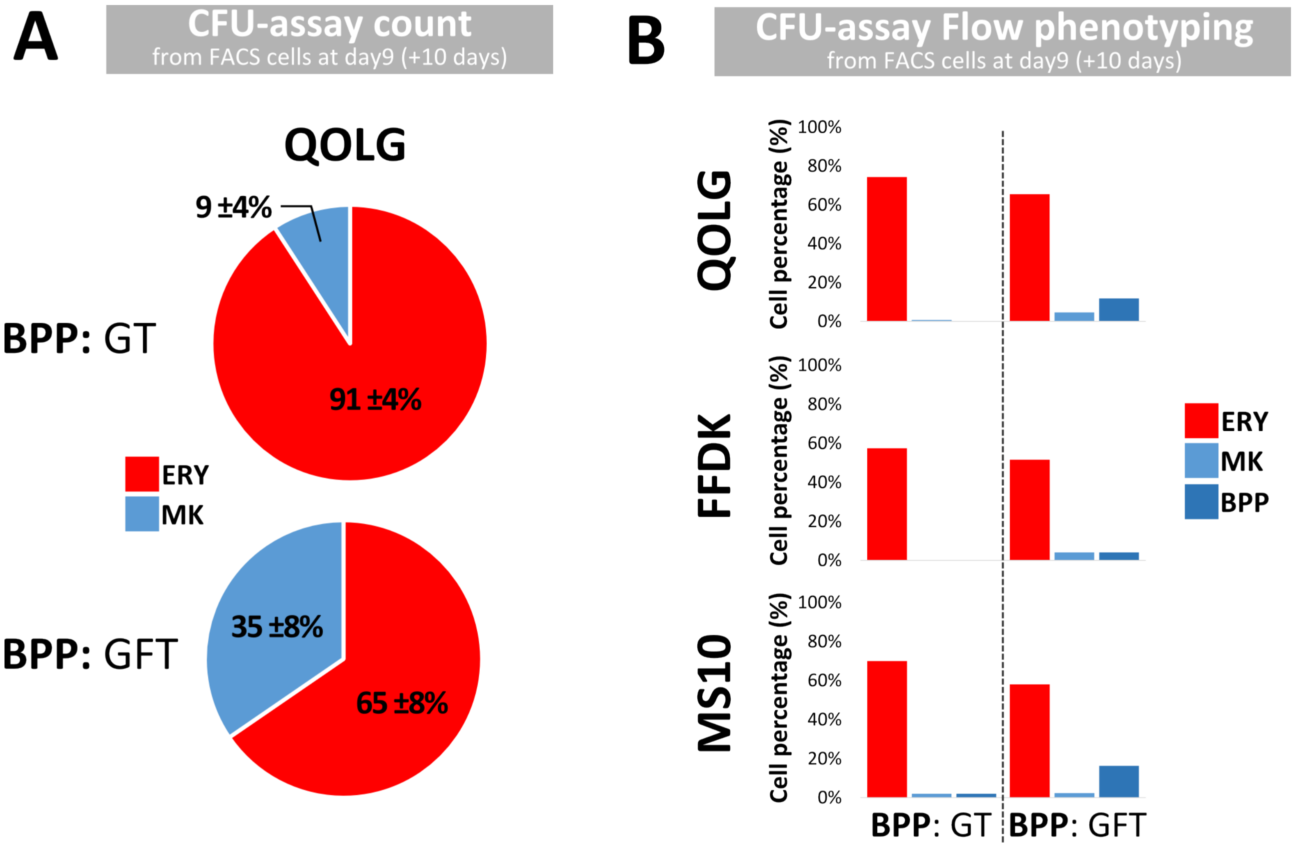

Supplementary Figure 5. Colony forming potential of day 9 BPPs sorted for GT or GFT transgene expression. Additional hPSC lines were forward programmed with rainbow vectors and the BPP population [CD41+/CD235+] was FACS sorted based on its expression of the GATA1+TAL1 [GT] or GATA1+FLI1+TAL1 [GFT] transgenes. The colony potential of each population was assessed in semi-solid methylcellulose clonogenic assays. The number, type (ERY/MK) and phenotype (CD41/CD235a) of colonies were determined after 10 days of culture as per Material & Methods. (**A**) Colony-type distribution after manual counting by 3 different operators of CFU-assay (as per Fig.1E) from biological duplicates of the QOLG cell line (mean +/-SD). (**B**) Phenotyping of the bulk colony assay content by flow cytometry. The total well content was collected and cells stained with CD41 and CD235a antibodies to define the ERY, BPP and MK populations as described in the main text (see Fig.1B). The distribution of the cell populations from 3 hPSC lines are shown (n=1).

Supplementary Figure 6. Expression time course of the haemogenic mesoderm marker CD309/FLK1 in forward programmed cells.

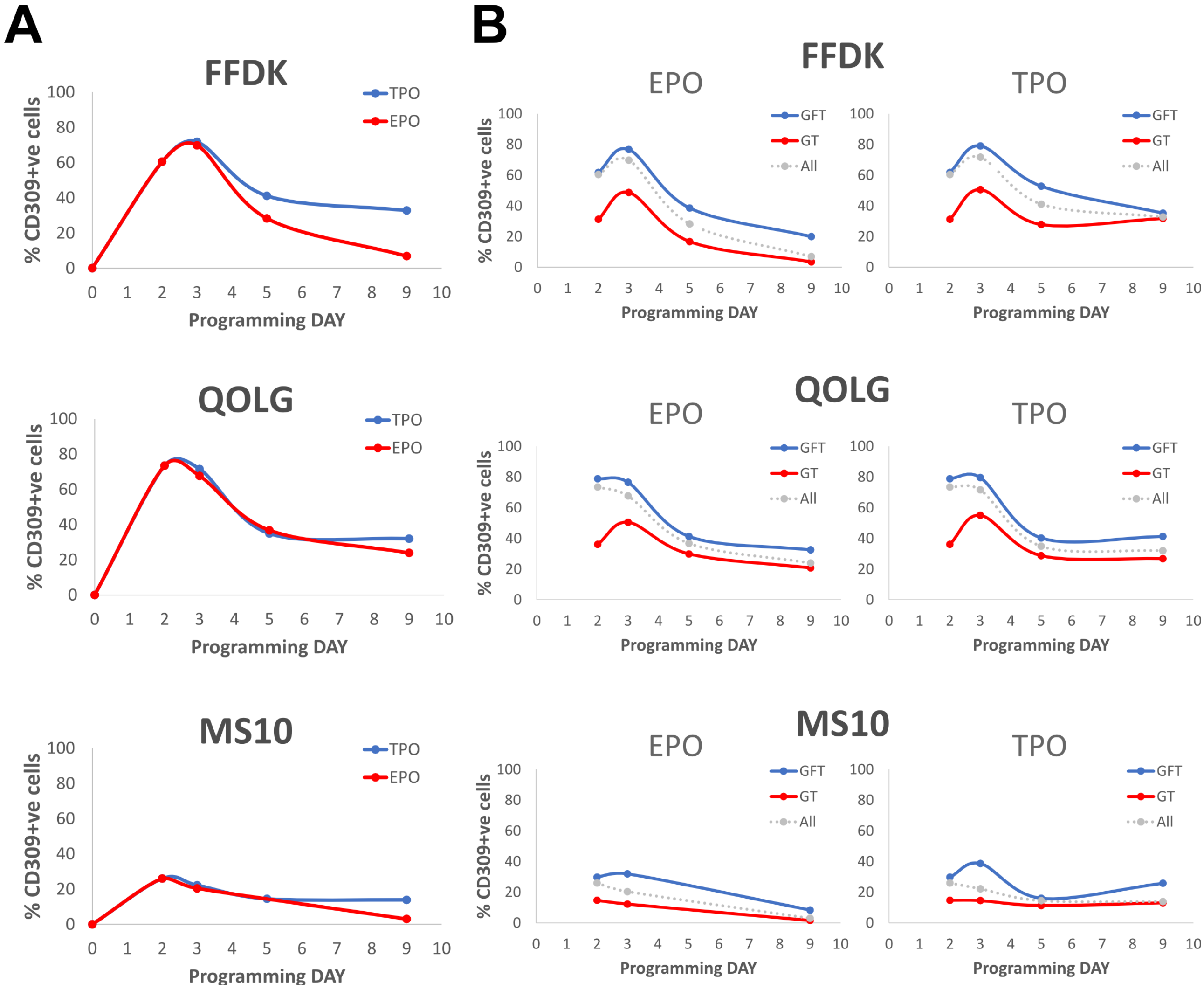

Supplementary Figure 6. Expression time course of the haemogenic mesoderm marker CD309/FLK1 in forward programmed cells. Three hPSC lines (FFDK, QOLG and MS10 lines) were programmed in TPO or EPO conditions with rainbow vectors and were analysed by flow cytometry between day 0-9 for the expression of rainbow transgenes and FLK1-APC. (**A**) The percentage of FLK1+ cells over time is shown for EPO (red) and TPO (blue) cultures for the 3 lines. (**B**) The percentage of FLK1-expressing cells was further analysed specifically inside the [GFT] and [GT] rainbow populations (dark and light blue respectively), vs. the whole live cell gate (grey dotted line).

Supplementary Experimental Procedure

Human pluripotent stem cell culture

The induced hPSC lines A1ATD1c (Yusa et al., 2011),FFDK_1, QOLG_3 (Kilpinen et al., 2017) and embryonic hPSC line MasterSheff-10 (UKSCB) (passage 30 to 50) were cultivated as clumps on feeder-free vitronectin coated wells (VTN-N; ThermoFisher) in a serum-free chemically defined medium (CDM) supplemented with recombinant zebrafish FGF2 and human Activin-A (15 ng ml^{-1} each; Cambridge Stem Cell Institute). The CDM was based on *Chen et al.* (Chen et al., 2011) and prepared with DMEM/F12-HEPES supplemented by 0.054% $NaHCO_3$, 20mg/L insulin, 11mg/L transferrin, 0.0134mg/L selenium (ThermoFisher) and 64mg/L L-Ascorbic Acid (Sigma Aldrich). All three iPSC lines have been derived from adult dermal fibroblasts using integrative murine retroviral vectors (A1ATD1c), episomal DNA vectors (FFDK_1) or non-integrative Sendai vectors (QOGL_3) for expression of the human OCT4, SOX2, KLF4 and MYC reprogramming factors. All cell cultures were maintained at 37°C and 5%CO_2 in humidified incubators.

Recombinant lentiviral vectors

The recombinant lentiviral vectors for expression of the three FoP factors have been previously described (Moreau et al., 2016) and are available at Addgene (ID.92415-7). Briefly, the coding sequences of variant-1 isoforms of GATA1, FLI1 and TAL1 (NM_002049.3, NM_002017.4 and NM_003189.5 NCBI Refseq, respectively) were inserted in place of eGFP into the 2nd generation replication deficient self-inactivating pWPT lentiviral backbone from the Trono lab (Addgene, ID.12255), driving ubiquitous transgene expression from the human EF1α promoter. To generate the rainbow vectors, reporter gene sequences (eGFP, dTomato and LSSmOrange) minus their stop-codon followed by the E2A self-cleaving peptide sequence were generated by PCR from plasmid templates. The obtained reporter-E2A fragments were individually cloned into the pWPT backbone replacing the eGFP. The three FoP factor-coding sequences were subsequently cloned in frame downstream the E2A using bespoke SalI/PspXI restriction sites. All recombinant constructs were sequence-verified.

VSV-G pseudotyped amphotropic lentiviral particles were produced by transient co-transfection of the recombinant vectors with the 2nd generation packaging plasmids pMD2.G and psPAX2 (Addgene, ID.12259-60, respectively) and concentrated 100-fold by PEG precipitation (Takara). Functional titres were determined by qPCR measurement of provirus copy number in genomic DNA or by flow cytometry detection of reporter gene in transduced HCT116 cells (ATCC CCL-247). Alternatively, lentiviral particles were produced commercially to >1E+9TU/mL as 3-vector mix (Vectalys). Transduction of hiPSC lines was performed by single exposure for 16-24 hours in a protamine sulphate-containing medium (10μg/mL) with multiplicity of infection (MOI) of 20 and 80 for each FoP vector and rainbow vector mix, respectively, leading to >80% transduction efficiencies.

Forward Programming

24 hours prior to transduction (day -1), sub-confluent (50–80%) hiPSC cultures were dissociated to small clumps using 0.5 mM EDTA DPBS, before seeding approximately 1E+5 cells onto one well of a vitronectin-coated 12 well plates. On transduction day (day 0), lentiviral particles were added to mesoderm medium -CDM supplemented with FGF2 (20 ng/mL), LY-294002 (10 μM, Sigma), BMP4 (10 ng/mL, Biotechne) and protamine sulphate (10 μg/mL). After 24 hours (day 1), cells were washed and fresh mesoderm media added without protamine sulphate. 24 hours later (day 2), culture media was changed to either erythroblast (ERY) or megakaryocyte (MK) medium, with half-volume renewal every 3 days. The ERY medium was composed of CellGro SCGM (CellGenix), EPO (2 U/mL, Biotechne), SCF (50 ng/mL, ThermoFisher), IL3 (10 ng/mL, Biotechne), insulin (20 μg/mL, Roche) and transferrin (60 μg/mL, Roche). The MK medium, CellGro SCGM, was supplemented with TPO (20 ng/mL, Biotechne) and SCF (25 ng/mL). At day 9 post-transduction, floating cells were collected from the supernatant and pooled with the adherent fraction of the culture dissociated to single cells using TrypLE (ThermoFisher). The single cell preparation was further cultivated in suspension at 2E+5 cells/mL on standard tissue culture vessels (Corning) for an additional 11 days in ERY or MK

medium as described above, including half medium renewal every 3 days and 1:4 cell split when cultures reached >1E+6 cells/mL.

Cord-blood Erythroblast Culture

CD34+ haematopoietic progenitors were isolated from human cord-blood samples obtained as part of an ethically approved study with informed consent by magnetic beads separation according to the manufacturer's protocol (Miltenyi Biotech Ltd). The cells were subsequently cultured in a three-stage protocol as previously described (Griffiths et al., 2012).

Flow cytometry analysis

Day 9 single-cell suspensions generated as above or day 20 cells directly collected from suspension cultures (up to 2E+5 cells) were stained for 20 min at room temperature in 50μL flow buffer (DPBS, 0.5% BSA, 2mM EDTA) using combinations of FITC, PE, PE-Cy7, APC and APC-H7 conjugated antibodies (Table S1), then washed with flow buffer once before analysis. Background fluorescences were set against fluorochrome-matched isotype control antibodies and compensation matrices defined using single-colour-stained cells. The rainbow reporter proteins were used in combination with PE-Cy7, APC and APC-H7 conjugated antibodies only, using single-reporter transduced cells for compensation calculation (Supplementary Figure 5). Flow count fluorospheres (Beckman Coulter) and DAPI (1µg/mL; Sigma Aldrich) were used to determine absolute viable cell count in samples. Enucleation was assessed by DRAQ5 (Biolegend) stain following manufacturer's instructions. Cells were co-stained with an anti-CD235a antibody to determine the percentage of CD235+ cells without nucleus. Flow cytometry experiments were performed on a Gallios flow cytometer and analysed using Kaluza 1.5a software (Beckman Coulter).

Colony forming unit assays

The clonogenic potential of FoP cultures was assessed in semi-solid methylcellulose medium supplemented with serum, EPO, SCF, IL-3, IL-6, G-CSF and GM-CSF (Stemcell Technologies, H4435). After flow cytometry analysis, single cell suspensions were seeded in 1.1mL containing 3.3E+3 cells plated in duplicate in high humidity chambers. The quantitative and qualitative colony outcome was monitored after 14 days upon blind microscopic observation from at least 2 people and results averaged. Flow cytometry analyses of colony assays were performed as above after picking individual colonies or dilution and collection of the whole culture wells in DPBS.

Gene expression analysis by RT-qPCR

Total RNA was extracted from pelleted cultured cells using RNeasy kits (Qiagen) according to the manufacturer's instructions and including DNase treatment. cDNA was prepared from 250–500 ng total RNA using Superscript III Reverse Transcriptase (ThermoFisher) according to the manufacturer's instructions and using random hexamers for priming. qPCR reactions were performed in duplicate using SYBR green chemistry on the ABI 7500HT or Mx3000P instruments (Applied Biosystems; Agilent Technologies). The relative gene expression was calculated by the $2^{-\Delta ct}$ method using GAPDH as housekeeping gene for normalisation. RT-qPCR primer pairs (Table S2) were designed to amplify only cDNA, detect all known isoforms and have no reported off-target matches against the human NCBI RefSeq database. All primers were tested before use for high PCR efficiencies and single dissociation curves. We used UTR priming (absent from transgene sequences) to specifically monitor endogene expression while transgene expression was selectively measured using a common reverse primer specific to the viral vector sequence.

Single cell RNA sequencing of rainbow sorted cells

Single cell library preparation. A forward programmed culture (TPO condition; day40: >99% CD41+, 76% CD42+) was single cell sorted by flow cytometry and 24 single cells from each of the 8 possible transgenic populations (from combination of the 3TFs) collected. The single cells were sorted in 96-well plates containing cell lysis buffer (0.2% Triton X-100 with RNase inhibitor) and frozen to -80°C before library preparation. The method for cDNA library making has been adapted from Smartseq2

for full-length RNAseq from single cells (Picelli et al. 2014). Briefly, the reverse transcription was initiated with oligo-dT primers using SuperScript-II reverse transcriptase and supplemented with ERCC RNA Spike-In (ERCC92, Thermo Fisher). The cDNA was then PCR amplified for 21 cycles using the KAPA HiFi Hotstart ReadyMix and IS primers (Kappa biosystems). The Nextera XT DNA chemistry (Illumina) was used to generate 96-cell indexed libraries. The sequencing was performed on an HiSeq-4000 system (Illumina; one 96-cell library per lane).

Single cell sequencing analysis. Raw sequencing reads (fastq files) were aligned to the human Ensembl reference genome GrCh38.81 supplemented with sequences for ERCC spike-in genes and the three fluorescent reporters eGFP, IssmOrange and dTomato expressed exogenously in FoP-MKs. Alignment was performed with GSNAP (Wu et al. 2010) with parameters *-n 1 -Q,* to discard multi-mapping reads. Unstranded uniquely mapped reads were then assigned to genes with HTSEQ-Count (Anders et al. 2015) with parameter *-s no*. Data was then quality-checked by discarding all cells with less than 400,000 read counts or showing high proportion of mitochondrial RNA reads (>10%) or ERCC spike-in reads (>2%), leaving 111/196 cells for further analyses. All non-detected genes (read count equal to zero for all cells) were removed. Read counts were then normalised with the scran algorithm (Lun et al., 2016) with parameters *min.sizes=20* and *sizes=10*. The sequences have been deposited with the following GEO accession number: GSE121579

Cell morphology

1-2E+5 single cells in suspension were spun onto a glass slide using cytofunnels at 400*g* for 5 minutes, methanol fixed, stained using the Giemsa (VWR) staining method and observed under phase contrast microscope.

Western blotting

Cells were lysed in RIPA buffer containing proteinase inhibitors (complete, Roche) and sonicated for 5 minutes. Samples were resolved by SDS-PAGE, transferred to a PVDF membrane (Millipore), blocked with 10% milk and incubated for 1 hour in specific antibodies for α, β, γ and ξ-globin and β-actin characterisation (table S1). Membranes were then incubated in specific HRP-conjugated secondary antibodies for 1 hour and bands were visualized using enhanced chemilunescence (G.E. Healthcare).

Statistics

Regression analysis.

The data for the Colony Forming Unit (CFU) experiments were analysed using mixed-effects Poisson regression in which the outcome was the number of colonies formed, after pooling counts within experimental duplicate. The model included a random effect corresponding to each technical operator and fixed effects representing replicate number, condition (i.e., cytokine for the first experiment and transcription factor combination for the second experiment) and an interaction between condition and cell type.

The flow cytometry data were analysed using mixed effects logistic regression. The binary outcome indicated whether a cell was CD41+CD235- (MK) or CD41+CD235+ (BPP) in one analysis and CD41-CD235+ (ERY) or CD41+CD235+ (BPP) in another analysis. The models included a fixed effect for each replicate and random slopes with common variance representing the replicate-specific changes in the log odds due to cytokine exposure (e.g. TPO vs EPO). The random slopes accounted for evident variation in the treatment effects across replicates (Supplementary Figures 3A-C).

The models for CFU and flow cytometry experiments were fitted using the glmer() function from the lme4 R package. Nested models fitted with the glmer() function were compared using the anova() R function.

General analysis.

Results are presented as mean ± SEM or SD, with *n* representing the number of biological replicates. P-values derived from applying either two-tailed Student's *t*-tests of a difference in means between

two samples or one-way ANOVA Tukey post-hoc test. These are mentioned in figure legends, where applicable. Statistical significance are indicated in the figures as follows: * for P-value<0.05, ** for P-value<0.01 and *** for P-value<0.001.

Vector maps

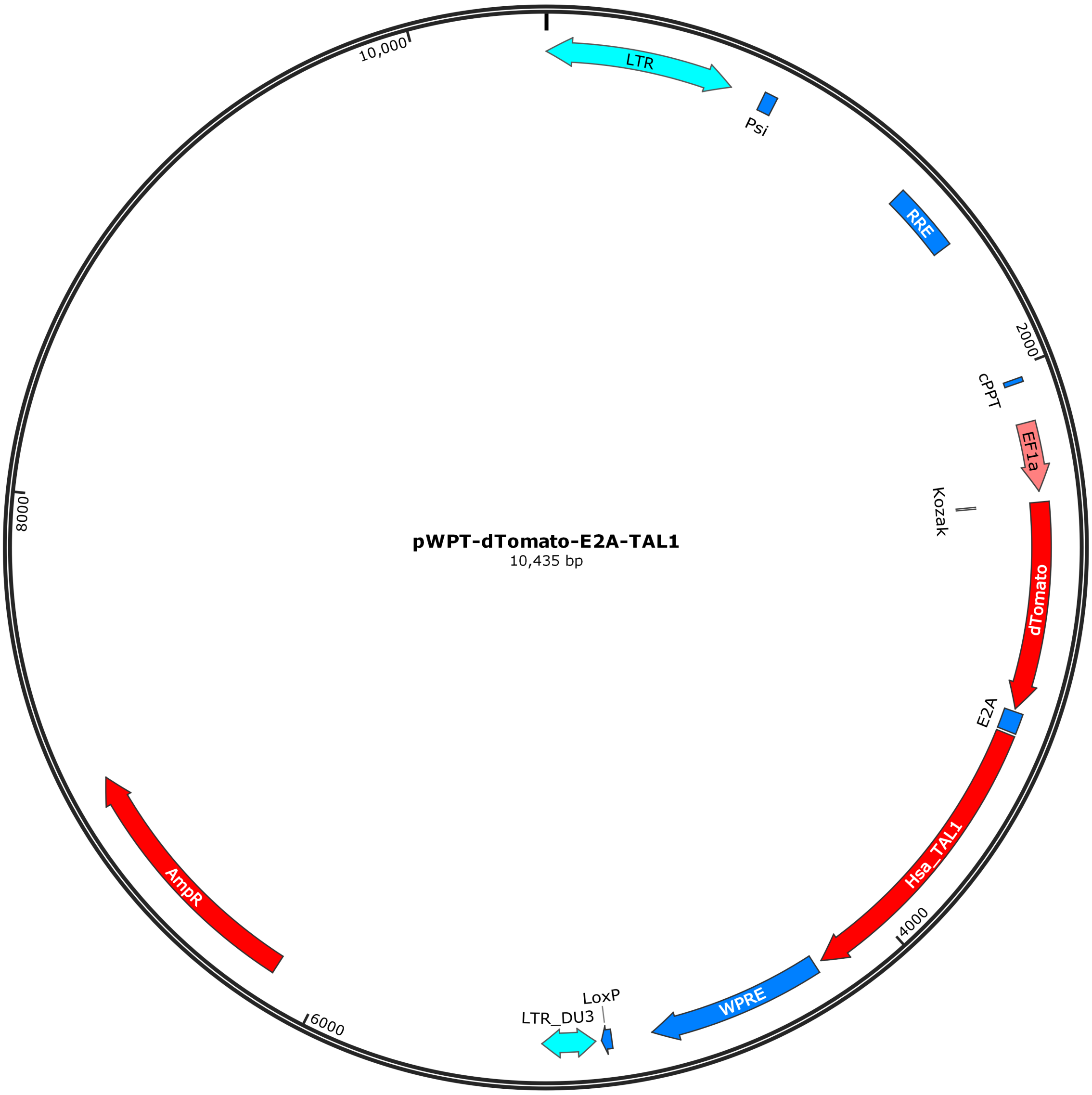

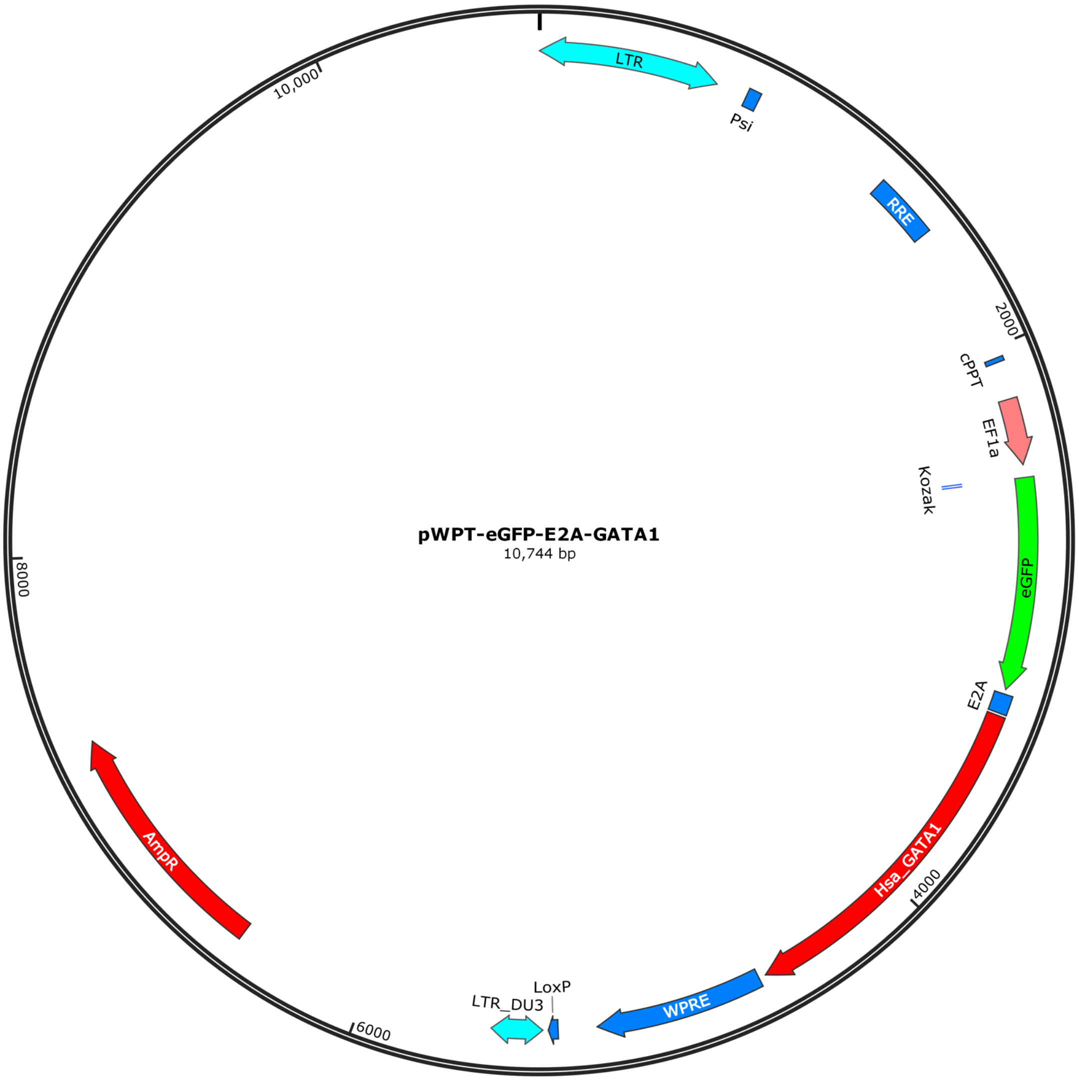
pWPT-eGFP-E2A-GATA1
10,744 bp
LTR
Psi
RRE
2000
cPPT
EF1a
Kozak
eGFP
E2A
Hsa_GATA1
4000
WPRE
LoxP
LTR_DU3
6000
AmpR
8000
10,000

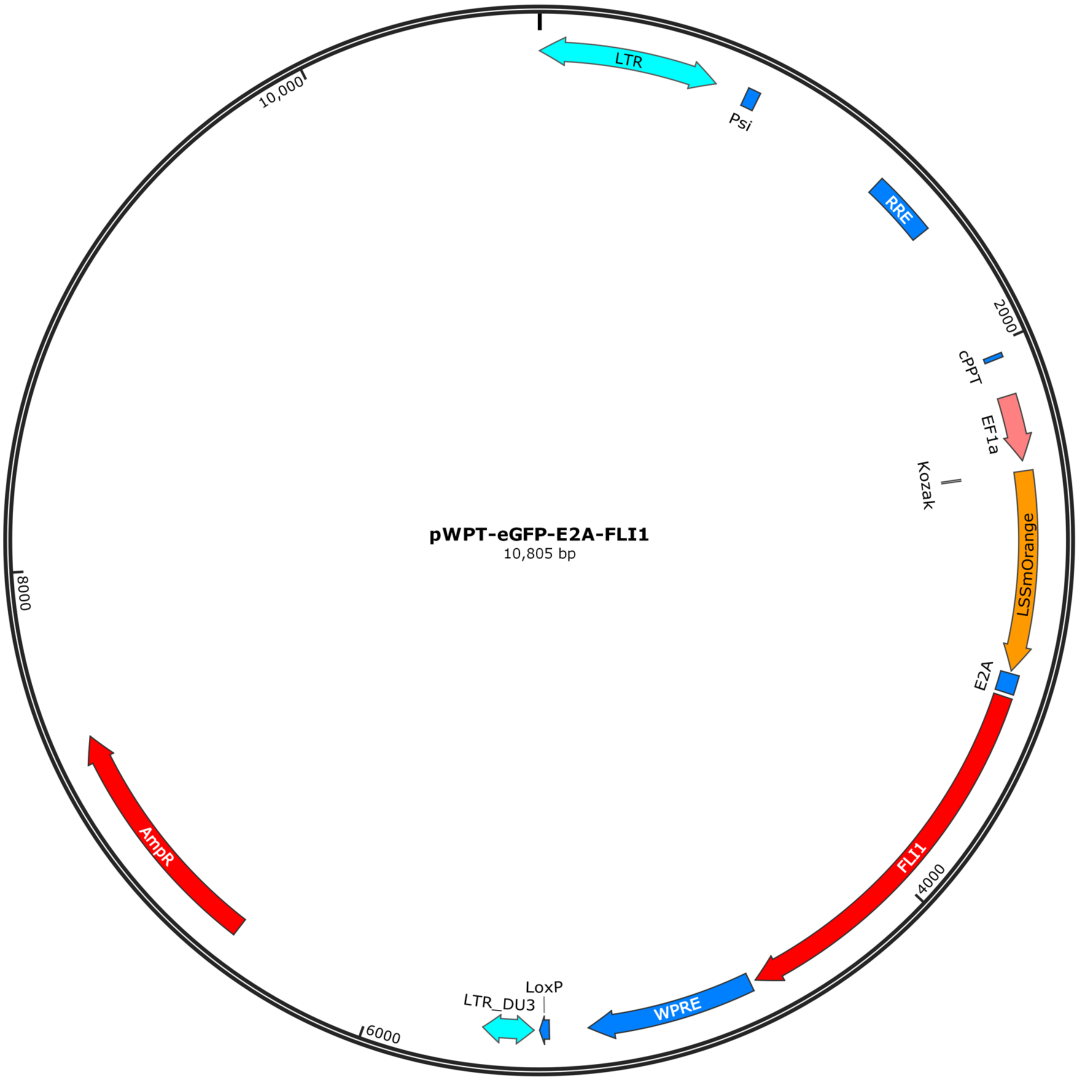
pWPT-eGFP-E2A-FLI1
10,805 bp
LTR
Psi
RRE
2000
cPPT
EF1a
Kozak
LSSmOrange
E2A
FLI1
4000
WPRE
LoxP
LTR_DU3
6000
8000
AmpR
10,000

Table S1. Antibodies used for flow cytometry analyses and western blotting.

Antigen	Antibody/Clone	Fluorochrome	Manufacturer
CD14	MφP9	FITC	BD Pharmingen
CD41a	559777	APC	BD Pharmingen
CD42a	558818	FITC	BD Pharmingen
CD66c	551478	PE	BD Pharmingen
CD71	M-A712	PE	BD Pharmingen
CD235a	555570	PE	BD Pharmingen
CD235a	GA-R2 (HIR2)	PE-Cy7	BD Pharmingen
BAND3	BRIC6-PE	PE	IBGRL Research Products
RhD	BRAD3-PE	PE	IBGRL Research Products
α-globin	sc-514378		Santa Cruz
β -globin	sc-21757		Santa Cruz
γ-globin	sc-21756		Santa Cruz
ξ-globin	ab156041		Abcam
β-actin	A5441		Sigma

Table S2. Oligonucleotides used in the study for qPCR analysis.

Target gene	Primer	Sequence (5'-3')
GATA1 (all)	Forward	CAGAACAGGCCCCTCATCC
GATA1 (all)	Reverse	TCAGTGGCCGGTTCACCT
GATA1 (TG)	Forward	GGTGGCTCCGCTCAGCTCAT
GATA1 (TG)	Reverse	GCAGCGTATCCACATAGCGTAAAAGG
GATA1 (endo)	Forward	TTGCCACATCCCCAAGGCGG
GATA1 (endo)	Reverse	GGGGGAGGGGCTCTGAGGTC
FLI1 (all)	Forward	ATACAACCTCCCACACCGAC
FLI1 (all)	Reverse	CTGATACGGATCTGGCTGGG
FLI1 (TG)	Forward	CCCGCCATCCTAACACCCACG
FLI1 (TG)	Reverse	GCAGCGTATCCACATAGCGTAAAAGG
FLI1 (endo)	Forward	GGGCTCGGCTGCAGACTTGG
FLI1 (endo)	Reverse	AGATGGGCTGCCGCTCCGTA
TAL1 (all)	Forward	GGAGACCTTCCCCCTATGAGA
TAL1 (all)	Reverse	CCCGGCTGTTGGTGAAGATA
TAL1 (TG)	Forward	AGGCGGTGGACTTGAACTTT
TAL1 (TG)	Reverse	TCTAGCCAGGCACAATCAGC
TAL1 (endo)	Forward	AGCAAAGACCCGGGTGTGCATC
TAL1 (endo)	Reverse	CCTCTAGCTGGGGGTCACTGCG
GAPDH	Forward	TATCGTGGAAGGACTCATGACC
GAPDH	Reverse	TAGAGGCAGGGATGATGTTCTG
KLF1	Forward	TGACTTCCTCAAGTGGTGGC
KLF1	Reverse	AGTTGGTGAGGAGGAGATCCA
HBB	Forward	TGGCAAGAAAGTGCTCGGTG
HBB	Reverse	CAGCTCACTCAGTGTGGCAAAG
HBE1	Forward	GGGCAGACTCCTCGTTGTTT
HBE1	Reverse	CCACATGCAGCTTGTCACAG
HBG	Forward	ATGGCAAGAAGGTGCTGACT
HBG	Reverse	TGGATTGCCAAAACGGTCAC
HBA	Forward	CCGACAAGACCAACGTCAAG
HBA	Reverse	GTCGAAGTGCGGGAAGTAGG
